# Central role of the proximal tubular αKlotho/FGF receptor complex in FGF23-regulated phosphate and vitamin D metabolism

**DOI:** 10.1038/s41598-018-25087-3

**Published:** 2018-05-02

**Authors:** Ai Takeshita, Kazuki Kawakami, Kenryo Furushima, Masayasu Miyajima, Kazushige Sakaguchi

**Affiliations:** 1 Department of Molecular Cell Biology and Molecular Medicine, Institute of Advanced Medicine, Wakayama Medical University, 811-1 Kimiidera, Wakayama, 641-8509 Japan; 2 Laboratory Animal Center, Wakayama Medical University, 811-1 Kimiidera, Wakayama, 641-8509 Japan

## Abstract

Fibroblast growth factor 23 (FGF23) plays critical roles in phosphate handling and vitamin D metabolism in the kidney. However, the effector cells of FGF23 in the kidney remain unclear. αKlotho, a putative enzyme possessing β-glucuronidase activity and also a permissive co-receptor for FGF23 to bind to FGF receptors (FGFRs), is expressed most abundantly in distal convoluted tubules, whereas it is expressed modestly in proximal tubules. Key molecular players of phosphate homeostasis and vitamin D-metabolizing enzymes are known to localize in proximal tubules. To clarify the direct function of FGF23 on proximal tubules, we ablated *αKlotho* or *Fgfr1–4* genes specifically from these tubules using the Cre-loxP-mediated genetic recombination. Both conditional knockout mouse lines showed similar phenotypes that resembled those of systemic *αKlotho* or *Fgf*23 knockout mice. Compared with control mice, they showed significantly elevated levels of plasma phosphate, FGF23 and 1,25-dihydroxyvitamin D, ectopic calcification in the kidney and aging-related phenotypes like growth retardation, osteoporosis and shortened lifespan. These findings suggest that the primary function of FGF23 on mineral metabolism is mediated through αKlotho/FGFR co-receptors expressed in proximal tubular cells, and that the putative enzymatic function of αKlotho in the proximal tubule has a minor role in systemic mineral metabolism.

## Introduction

αKlotho was discovered initially as a molecule related to aging^1^. Mice in which this gene is deleted show signs of early aging, such as vascular calcification, osteopenia, skin atrophy, ectopic calcification, pulmonary emphysema, growth retardation, and shortened lifespan. The biochemical phenotypes of these mice include hyperphosphatemia, hypercalcemia, low levels of parathyroid hormone (PTH), and increased levels of fibroblast growth factor 23 (FGF23) and 1,25-dihydroxyvitamin D (1,25(OH)_2_D)^2^. αKlotho has β-glucuronidase activity^3^ and also acts as a permissive co-receptor for FGF23 to bind to FGF receptors (FGFRs)^4^. The somatic features of *αKlotho* knockout (KO) mice are reminiscent of those of *Fgf23* KO mice^5^.

FGF23 plays pivotal roles in the regulation of phosphate and vitamin D metabolism. Phosphate is absorbed through the intestine, stored in bone, and excreted into urine. Renal reabsorption from proximal tubules is one of the key steps in maintaining phosphate homeostasis in mammals. FGF23 inhibits renal phosphate reabsorption by internalizing the sodium-dependent phosphate co-transporters NPT2A and NPT2C^6–8^. Vitamin D requires an activation step in proximal tubules of the kidney to generate 1,25(OH)_2_D via 1α-hydroxylation by CYP27B1. Vitamin D is inactivated via 24-hydroxylation by CYP24A1 in the same proximal tubules. These vitamin D regulation processes are also under the tight control of FGF23 through the suppression of CYP27B1 and upregulation of CYP24A1^9^. Although proximal tubules are the functional domain for phosphate handling and vitamin D activation, there are two conflicting reports concerning the target tubules for FGF23-mediated phosphaturic effect and vitamin D metabolism in the kidney, namely, distal and proximal tubules^10,11^. If distal tubules are the target, we would have to hypothesize a distal to proximal feedback mechanism^12^.

A few studies have been performed to locate the tubules in the kidney that are responsible for FGF23 function using the tubule-specific ablation of the *αKlotho* gene. In the kidney, αKlotho expression is highest in distal tubules, while it is modest in proximal tubules^13,14^. One study group examined the effect of αKlotho deletion from distal tubules of *αKlotho*^*flox*/*flox*^ mice using *Ksp-cadherin-Cre* transgenic mice^15^, in which the authors assumed that Cre is expressed specifically in distal tubules. In these transgenic mice, however, Cre is clearly shown to be expressed in almost all epithelial cells including those in proximal tubules though the proximal tubular expression is low^16^. The original characterization report of these transgenic mice showed that recombination efficiency is not homogeneous even in the tubules with high levels of Cre expression^16^. By mating *αKlotho*^*flox*/*flox*^ mice with these mice, they found mild hyperphosphatemia, modestly elevated serum FGF23 levels, decreased serum levels of PTH, and the abundant expression of the sodium-phosphate co-transporter NPT2A at the brush border membrane. However, *Ksp-cadherin-Cre;αKlotho*^*flox*/*flox*^ mice had a normal gross phenotype and normal levels of serum calcium and 1,25(OH)_2_D. These phenotypes appear to be inconsistent with the results reported by others^17,18^.

The same group also ablated αKlotho from proximal tubules^19^. They mated *αKlotho*^*flox*/*flox*^ mice with *Cre* transgenic mice expressing Cre recombinase under the control of three different inducible promoters (*PEPCK-Cre*, *Kap-Cre*, and *Slc3*4*a1-Cre*)^20–22^. These three different *Cre-αKlotho flox* mouse lines showed mild or no hyperphosphatemia under basal dietary conditions. The effects on 1,25(OH)_2_D and FGF23 varied among these mouse lines, but were modest overall and not statistically significant. They did not show any gross phenotypes.

These reported studies demonstrated equivocal effects of αKlotho expressed in either proximal or distal tubules. In addition, they did not answer whether the effect of αKlotho is limited to modulation of FGF23-FGFR interactions, since αKlotho is also known to function independently of FGFRs. αKlotho has β-glucuronidase activity and directly inhibits the transporter activity of NPT2A in the proximal tubule urinary lumen by the modification of glycans and proteolytic degradation of NPT2A^13^.

Therefore, we examined the effect of the proximal tubule-specific ablation of *αKlotho* and *Fgfr1–4* (*Fgfr1*, *2*, *3*, and *4*) genes on mineral metabolism using *αKlotho*^*flox*/*flox*^ or *Fgfr1–4*^*flox*/*flox*^ mice^23^ mated with a distinct Cre-expression mouse strain, *Ndrg1-CreERT2* transgenic mice^24^, which expresses Cre in proximal tubules upon tamoxifen treatment. Our findings in these mice reproduced those of systemic *αKlotho* KO mice for lifespan and both biochemical and skeletal phenotypes.

## Results

### Specific expression of Ndrg1-CreERT2 in proximal tubules

**Figure 1:**
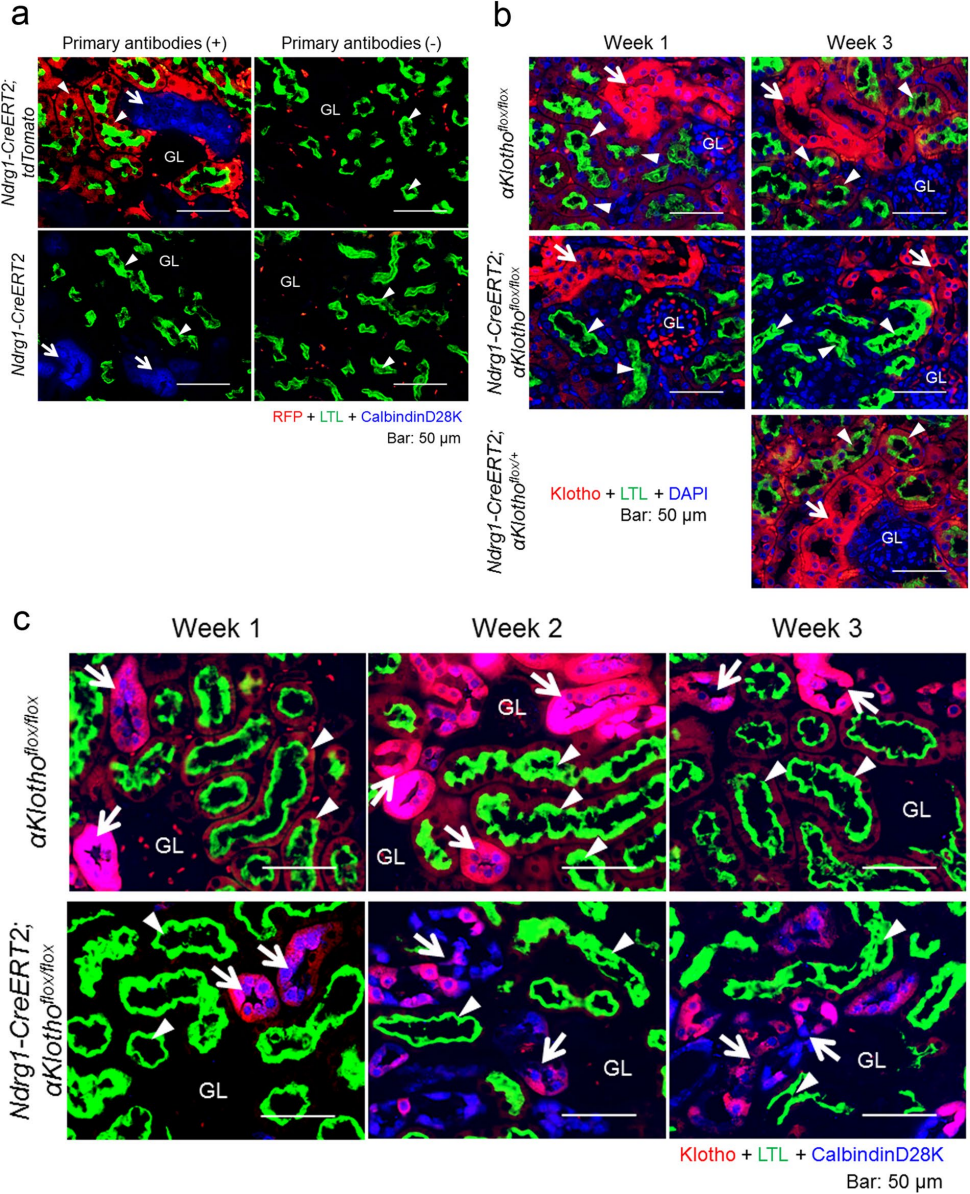
Proximal tubule-specific expression of Ndrg1-CreERT2 and its recombination efficiency in *Ndrg1-CreERT2;αKlotho*^*flox*/*flox*^ mice. (**a**) Proximal tubule-specific expression of Cre in *B6.Cg-Gt(ROSA)26Sor*^*tm9(CAG-tdTomato)Hze*^/*J*(*tdTomato*) mice mated with *Ndrg1-CreERT2* mice. Tamoxifen (3 mg/20 g body weight/day) was injected intraperitoneally for 5 consecutive days at the age of 5–8 weeks, and the mice were sacrificed 3 weeks later. Left panels: Kidney sections were co-immunostained for red fluorescent protein (RFP; red) and calbindinD28K (a marker for distal convoluted tubules shown in blue, arrows) with affinity marking by FITC-conjugated lotus tetragonolobus lectin (LTL, green) of the brush border membrane of proximal tubules (arrowheads). Right panels: The same experiments without any primary antibody (anti-RFP or anti-calbindinD28K antibody). Cre expression shown by positive RFP staining was specific for proximal tubules. GL: glomerulus. Scale bars: 50 μm. (**b**) Renal αKlotho expression at 1 and 3 weeks after tamoxifen treatment in *αKlotho*^*flox*/*flox*^, *Ndrg1-CreERT2;αKlotho*^*flox*/*flox*^, and *Ndrg1-CreERT2;αKlotho*^*flox*/*+*^ mice. Tamoxifen was injected to induce Cre as described above. The period after tamoxifen treatment was counted from the date of the initial tamoxifen injection. Kidney sections were immunostained for αKlotho (red) and LTL (green), and counterstained for cell nuclei (DAPI, blue). Arrows, distal convoluted tubules; arrowheads, proximal tubules. Scale bars: 50 μm. (**c**) Co-staining of αKlotho and calbindinD28K in the kidney cortex of *αKlotho*^*flox*/*flox*^ and *Ndrg1-CreERT2;αKlotho*^*flox*/*flox*^ mice. Kidney sections treated as in (**b**) were stained for αKlotho (red), LTL (green), and calbindinD28K (blue). αKlotho expression was almost completely abolished in proximal tubules at 1 week after the initial tamoxifen injection. Distal convoluted tubules started to disassemble and their αKlotho expression intensity began to decrease at 2 weeks after tamoxifen injection. Arrows, distal convoluted tubules; arrowheads, proximal tubules. Scale bars: 50 μm. These experiments were performed in paired littermates with and without *Ndrg1-CreERT2 transgene* under the C57BL/6 J *αKlotho*^*flox*/*flox*^ background. Similar findings were reproduced in at least 3 different litters. See also Supplementary Figures S1 and S2.

The specificity of Ndrg1-CreERT2 expression in the kidney tubules using *Rosa26ECFP* mice has been reported previously^24^. Recombination efficiencies in proximal tubules, distal tubules, and collecting ducts were 90% (100% in the S1 and S2 segments and 58% in the S3 segment), 4%, and 32%, respectively. In addition to these studies, we examined *Ndrg1-CreERT2* mice crossed with B6.Cg-*Gt(ROSA)26Sor*^*tm9(CAG-tdTomato)Hze*^/J (*tdTomato*) mice (Jackson Laboratory, Bar Harbor, ME) at 3 weeks after intraperitoneal injection of tamoxifen (3 mg/20 g body weight/day) to 5–8 week old mice for 5 consecutive days. As shown in Fig. 1a, we stained kidney sections taken from the mice for red fluorescent protein (RFP) expression together with markers for proximal and distal convoluted tubules, lotus tetragonolobus lectin (LTL) and CalbindinD28K, respectively. Cell nuclei were counter-stained with DAPI. The number of RFP + cells was counted in 75 LTL + proximal tubules (610 cells) and in 40 calbindinD28K + distal convoluted tubules (279 cells) located in the cortex. RFP was positive in 98.2% of LTL + proximal tubular cells, but in only 0.4% of calbindinD28K + distal tubular cells.

### Tamoxifen treatment of *Ndrg1-CreERT2;αKlotho*^*flox*/*flox*^ mice induces an early decrease of αKlotho expression in proximal tubules and a subsequent decrease in distal tubules

**Figure 2:**
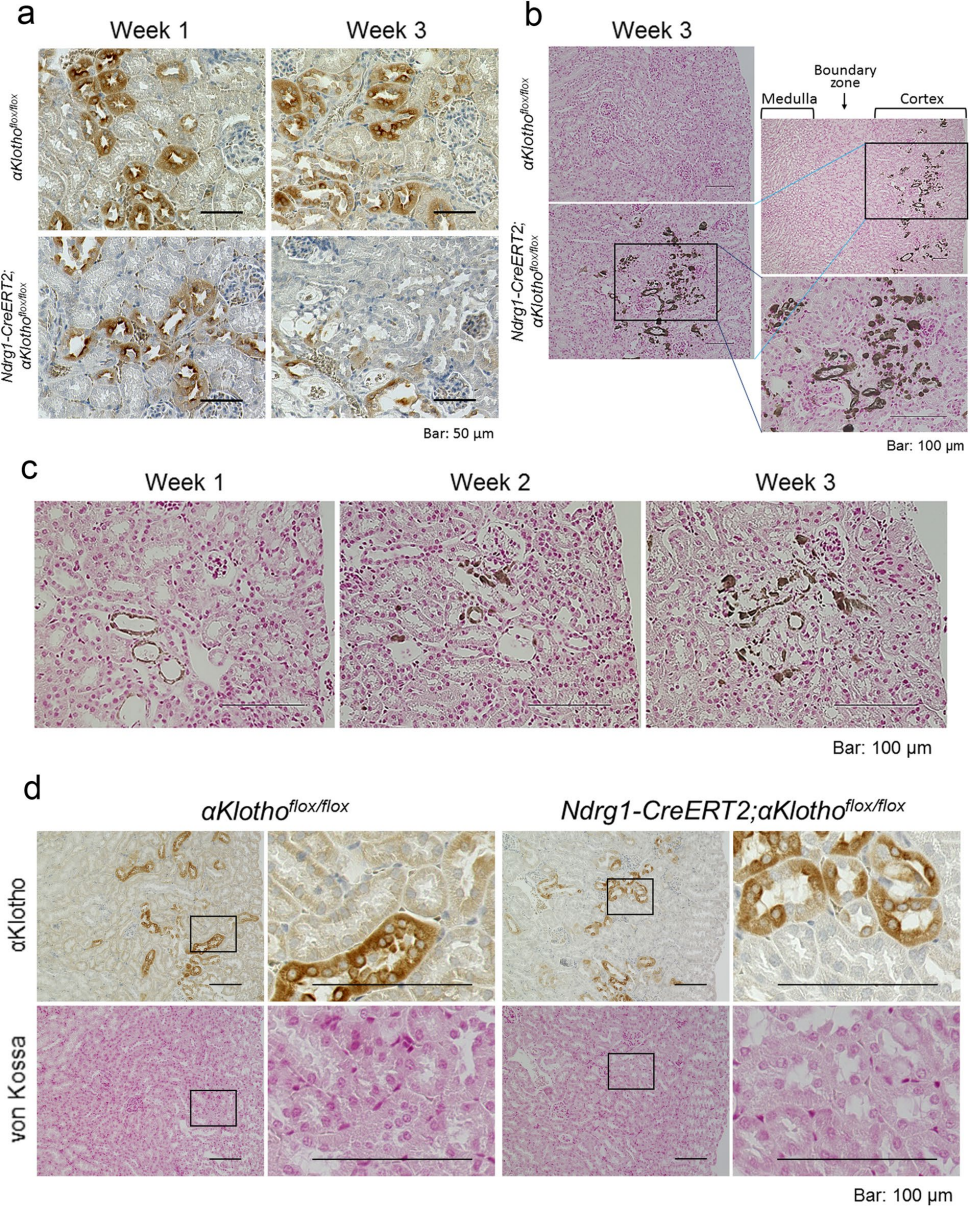
Proximal tubule-specific *αKlotho* cKO induces ectopic calcification in the kidney cortex and reduces αKlotho expression in distal tubules, both of which can be reversed by a vitamin D-deficient diet. (**a**) αKlotho expression in the kidney of *αKlotho*^*flox*/*flox*^ and *Ndrg1-CreERT2;αKlotho*^*flox*/*flox*^ mice 1 and 3 weeks after tamoxifen treatment. Kidney sections were stained for αKlotho using DAB with counterstaining by hematoxylin. At 1 week after tamoxifen treatment, αKlotho staining was significantly reduced in proximal tubules, whereas distal convoluted tubules retained αKlotho expression. At 3 weeks after tamoxifen treatment, non-stained (by either DAB or hematoxylin) empty areas became evident in the cortex, and αKlotho expression was reduced dramatically in distal convoluted tubules in addition to its deleted expression in proximal tubules. Kidney cortical surface is oriented to the right. Scale bars: 50 μm. (**b**) Renal ectopic calcification. Kidney sections from mice treated with tamoxifen for 3 weeks were stained for calcified tissue (dark brown) using the von Kossa method. *Ndrg1-CreERT2;αKlotho*^*flox*/*flox*^ mice showed marked calcification in only the cortex, whereas *αKlotho*^*flox*/*flox*^ mice did not exhibit any calcification in the kidney. Shown on the right are the wider (top) and the magnified (bottom) views of the calcified area marked by a rectangle. Scale bars: 100 μm. (**c**) Time course of renal calcification after tamoxifen treatment. Kidney sections from *Ndrg1-CreERT2;αKlotho*^*flox*/*flox*^ mice were stained for renal calcification using the Von Kossa staining method. The calcified area increased in a time-dependent fashion after tamoxifen treatment. (**d**) Alleviation of renal calcification by a vitamin D-deficient diet. Vitamin D-deficient diet was initiated after confirming pregnancy and continued thereafter until sacrifice of the offspring. Mice were sacrificed at the age of 9 weeks and at 3 weeks after tamoxifen treatment. Kidney serial sections were stained for αKlotho and calcification using the standard DAB method and von Kossa staining technique, respectively. Vitamin D-deficient diet nullified ectopic renal calcification and restored αKlotho expression in distal tubules. Shown on the right are the magnified views of the cortical area marked by rectangles on the left. Scale bars: 100 μm. All of the above results were reproduced in 3 different pairs of littermates.

We then examined the expression of αKlotho in renal tubules after the induction of Ndrg1-Cre by intraperitoneal injections of tamoxifen (3 mg/20 g body weight/day) for 5 consecutive days. In *αKlotho*^*flox*/*flox*^ mice and control heterozygous flox mice, *Ndrg1-CreERT2;αKlotho*^*flox*/*+*^ mice, αKlotho was expressed abundantly, even after tamoxifen injection, in distal convoluted tubules (marked by calbindinD28K) and less abundantly in proximal tubules (labeled with LTL) (Fig. 1b). However, *Ndrg1-CreERT2;αKlotho*^*flox*/*flox*^ mice lost αKlotho expression almost completely in proximal tubules at 1 week after the initial tamoxifen injection (Fig. 1b and c). The expression of αKlotho in distal tubules was as intense as in control mice at 1 week after tamoxifen injection, whereas distal tubules appeared to disassemble morphologically and αKlotho expression apparently decreased at 2 and 3 weeks after injection (Fig. 1b and c). We also detected the morphological disassembly by hematoxylin and eosin (HE) staining of kidney sections (Supplementary Figure S1). The number of aquaporin 2-positive collecting tubule cells that were located around both the kidney cortex and corticomedullary boundary areas and their αKlotho expression intensity remained unchanged at 3 weeks after injection (Supplementary Figure S2a and b). Furthermore, connecting tubules, which are located between distal convoluted tubules and collecting ducts and labeled by both calbindinD28K and aquaporin 2, retained their morphological structure and αKlotho expression, even at 3 weeks after tamoxifen injection (Supplementary Figure S3). Supplementary Figure S3 also shows morphological disassembly and decreased αKlotho expression in distal convoluted tubules. The detrimental effect of αKlotho conditional KO (cKO) from proximal tubules on distal tubular cells was also confirmed in kidney sections stained for αKlotho with DAB (Fig. 2a). At 1 week after the initial tamoxifen injection, in *Ndrg1-CreERT2;αKlotho*^*flox*/*flox*^ mice, αKlotho expression was lost from proximal tubules, while distal tubules retained αKlotho expression. However, at 3 weeks after the initial tamoxifen injection, the intensity of αKlotho expression was reduced from not only proximal tubules but also distal tubules. Furthermore, the cortical tubular cells surrounding the glomeruli and vasculature appeared disassembled after 3 weeks.

### Proximal tubule-specific *αKlotho* cKO induces ectopic calcification in the kidney cortex, which can be reversed by a vitamin D-deficient diet

**Figure 3:**
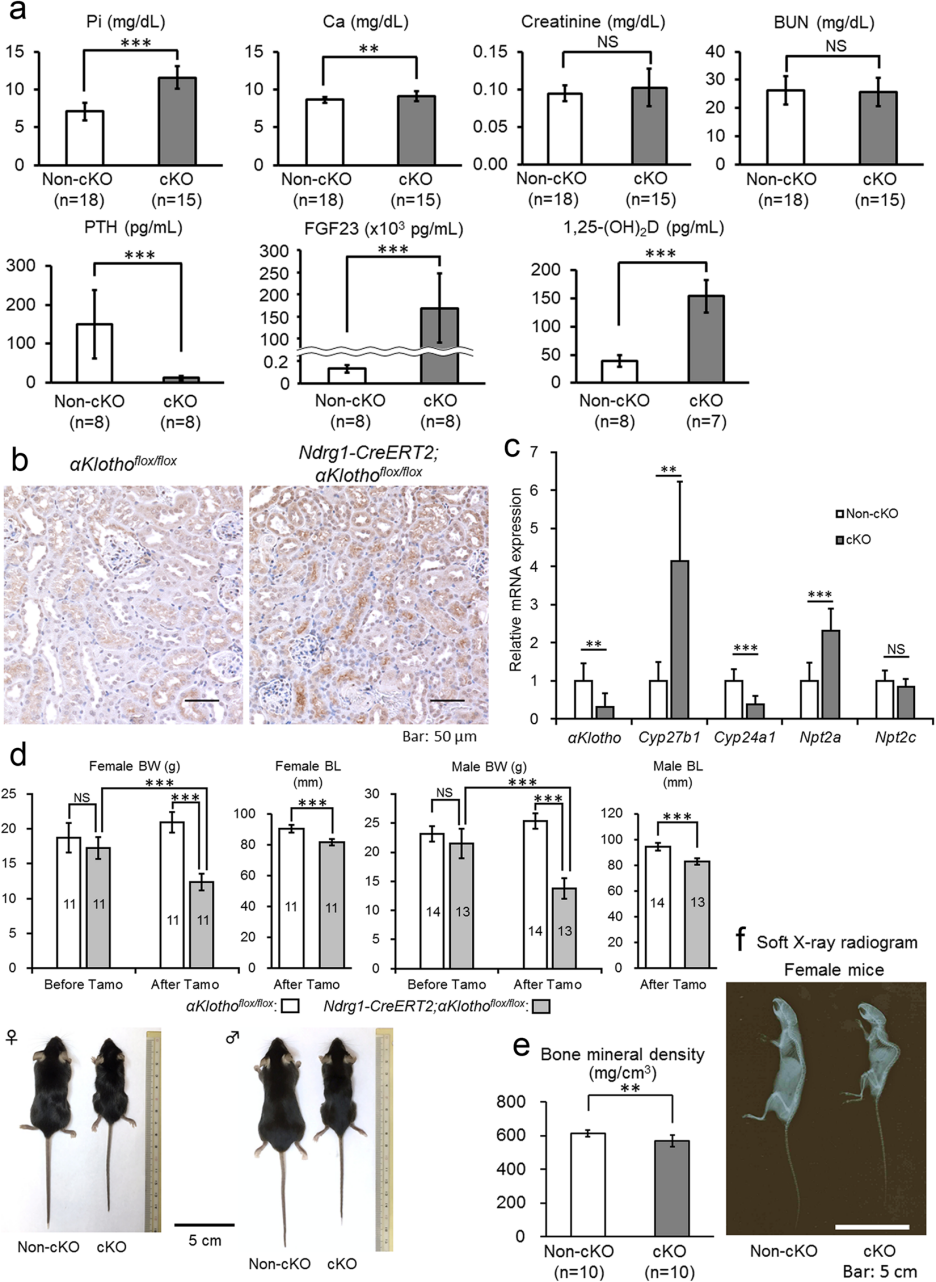
Proximal tubule-specific *αKlotho* cKO reproduces the phenotypes of systemic *αKlotho* KO. (**a**) Biochemical analysis of plasma inorganic phosphate (Pi), calcium (Ca), creatinine, blood urea nitrogen (BUN), parathyroid hormone (PTH), fibroblast growth factor 23 (FGF23), and 1,25-dihydroxyvitamin D (1,25(OH)_2_D) in *αKlotho*^*flox*/*flox*^ (non-cKO) and *Ndrg1-CreERT2;αKlotho*^*flox*/*flox*^ (cKO) mice at 9 weeks of age at 3 weeks after the induction of Cre by tamoxifen treatment. These parameters were measured as described in the Methods section. Values are expressed as mean ± s.d. Number of samples (n) is described in the figure. (**b**) Expression of NPT2A in the renal cortex. Kidney sections were immunostained for NPT2A with the standard DAB method. (**c**) Reverse transcription quantitative polymerase chain reaction (RT-qPCR) of RNA extracted from the kidney. RT-qPCR was performed as described in the Methods section. Primers used for these studies are listed in Supplementary Table S4. Values are expressed as mean ± s.d. of 7 samples for non-cKO mice and 8 samples for cKO mice. (**d**) Mouse body size. Body weight (BW) was measured at 6 weeks of age before Cre induction and at 9 weeks of age at 3 weeks after Cre induction, whereas body length (BL) was measured only at 3 weeks after Cre induction. Data show a body size reduction in *αKlotho* cKO mice as compared with non-cKO control mice. Values are expressed as mean ± s.d. Number of animals used in each group is indicated in each bar graph. (**e**) Bone analysis. Bone mineral density of the femurs was analysed with micro-CT as described in the Methods section. Values are expressed as mean ± s.d. of 10 samples. f. Soft X-ray radiogram of representative non-cKO and cKO mice after tamoxifen treatment. Statistical significance between 2 groups was examined by a two-tailed unpaired Student’s t test, whereas the significance among more than 3 groups was analysed by one-way ANOVA followed by Tukey’s multiple comparison test. NS, not significantly different; *P < 0.05; **P < 0.01; ***P < 0.001. See also Supplementary Tables S1 and S2.

In systemic *αKlotho* KO mice, ectopic calcification is prevalent throughout the body. We examined calcification in the kidney during this proximal tubule-specific KO process to identify the cause of damage to distal tubules. Surprisingly, in *Ndrg1-CreERT2;αKlotho*^*flox*/*flox*^ mice at 3 weeks after tamoxifen injection, we found ectopic calcification in only the cortical area where glomeruli, vasculature, and distal convoluted tubules were abundant (Fig. 2b). Proximal tubules located near the surface of the kidney were almost completely spared from calcification, and the corticomedullary boundary zone and the medulla area were completely free of calcification. *αKlotho*^*flox*/*flox*^ mice used as a control after tamoxifen injection were completely free of ectopic calcification. We also examined the time course of intra-renal ectopic calcification in *Ndrg1-CreERT2;αKlotho*^*flox*/*flox*^ mice for up to 3 weeks after injection (Fig. 2c). Ectopic calcification was minimal at 1 week after the first tamoxifen injection, whereas calcification increased gradually thereafter. Vitamin D-deficient diet is known to reverse the renal calcification in systemic *αKlotho* KO mice^2^. We, therefore, examined whether this diet can alleviate the kidney calcification in our *αKlotho* cKO mice (Fig. 2d). Vitamin D-deficient diet reduced the level of 1,25(OH)_2_D (cKO vs. non-cKO mice: 11.9 ± 2.7 [n = 4] vs. 5.2 ± 2.3 pg/mL [n = 7]) as compared with the mice on a regular diet (cKO vs. non-cKO mice: 154.3 ± 28.9 pg/mL [n = 7] vs. 38.7 ± 10.3 pg/mL [n = 8]; see also Fig. 3a) and induced null calcification in the kidney. *αKlotho* expression was lost in proximal tubules but retained in distal tubules, confirming the importance of active vitamin D in renal calcification and suggesting renal calcification as a cause of decreased αKlotho expression in distal tubules. FGF23 stayed high in the absence of renal calcification (cKO vs. non-cKO mice: 63437 ± 40228 [n = 4] vs. 93 ± 36 pg/mL [n = 7]), implying that FGF23 plays a minor role in renal calcification.

### Proximal tubule-specific *αKlotho* cKO mice shows phenotypes similar to those of systemic *αKlotho* KO mice

The *αKlotho*-cKO mice showed plasma levels of biochemical and humoral factors that were similar to those of the systemic KO mice in comparison with non-cKO control mice, *αKlotho*^*flox*/*flox*^: elevated phosphate (cKO mice vs. non-cKO mice: 11.61 ± 1.51 mg/dL [n = 18] vs. 7.13 ± 1.14 mg/dL [n = 15], *P* = 6.5 × 10^−11^), calcium (9.18 ± 0.63 mg/dL [n = 18] vs. 8.66 ± 0.44 mg/dL [n = 15], *P* = 0.0093), FGF23 (169,133 ± 77,972 pg/mL [n = 8] vs. 130 ± 34 pg/mL [n = 8], *P* = 0.000026), and 1,25(OH)_2_D (154.3 ± 28.9 pg/mL [n = 7] vs. 38.7 ± 10.3 pg/mL [n = 8], *P* = 0.000015) levels, and decreased PTH (12.67 ± 4.95 pg/mL [n = 8] vs. 150.48 ± 87.69 pg/mL [n = 8], *P* = 0.0015) levels (Fig. 3a). The parameters of renal function were not significantly different between cKO and non-cKO mice: creatinine (cKO mice vs. non-cKO mice: 0.102 ± 0.025 [n = 18] vs. 0.094 ± 0.010 [n = 15], *P* = 0.29) and blood urea nitrogen (BUN; 25.6 ± 5.1 [n = 18] vs. 26.4 ± 5.0 [n = 15], *P* = 0.66). *αKlotho*^*flox*/*flox*^ and *αKlotho*^*flox*/*flox*^*;Ndrg1-CreERT2* mice without tamoxifen treatment showed no difference in these parameters (Supplementary Table S1).

We examined the expression of NPT2A in proximal tubular cells using immunohistochemical staining. Proximal tubular cells from tamoxifen-injected *Ndrg1-Cre;αKlotho*^*flox*/*flox*^ mice exhibited high levels of NPT2A expression, especially at the apical side, whereas the control mice, *αKlotho*^*flox*/*flox*^, showed very low expression levels of this Na-Pi cotransporter (Fig. 3b).

We also quantified the mRNA expression of several molecules in the kidney using reverse transcription-quantitative polymerase chain reaction (RT-qPCR) with *glyceraldehyde-3-phosphate dehydrogenase* (*Gapdh*) as a reference gene to calibrate their expression. When compared with control animals (*αKlotho*^*flox*/*flox*^) (n = 7) after tamoxifen injection, *Ndrg1-Cre;αKlotho*^*flox*/*flox*^ mice (n = 8) showed decreased mRNA expression of *αKlotho* (by 69%, *P* = 0.0068) and *Cyp24a1* (by 61%, *P* = 0.00051) and increased mRNA expression of *Cyp27b1* (4.2-fold, *P* = 0.0019) and *Npt2a* (2.3-fold, *P* = 0.00036) (Fig. 3c). *Npt2c* mRNA expression did not change significantly (*P* = 0.23). These findings are consistent with the reported effect of *Fgf23* or *αKlotho* KO on the enzymes related to phosphate and vitamin D metabolism^7,9,25^.

*αKlotho*^*flox*/*flox*^ and *αKlotho*^*flox*/*flox*^*;Ndrg1-CreERT2* mice did not show any statistically significant difference in body weight before tamoxifen treatment. However, after tamoxifen treatment *αKlotho*-cKO mice showed a significant reduction in body size compared with control mice: female body weight, 12.4 ± 1.2 g (n = 11) vs. 20.9 ± 1.5 g (n = 11), *P* < 0.001; male body weight, 13.7 ± 1.7 g (n = 11) vs. 25.3 ± 1.3 g (n = 11), *P* < 0.001; female nose-to-anus length, 81.5 ± 2.2 mm (n = 11) vs. 90.2 ± 2.4 mm (n = 11), *P* < 0.001; male nose-to-anus length, 83.0 ± 2.6 mm (n = 11) vs. 94.4 ± 2.9 mm (n = 11), *P* < 0.001, respectively (Fig. 3d). We also analysed bone mineral density of the entire femur using micro-computed tomography (CT) (Fig. 3e) and found that bone mineral density was significantly decreased in the mutants. Mutant mice showed the typical kyphosis seen in systemic *αKlotho* KO mice (Fig. 3f).

We also examined the survival of 12 *αKlotho* cKO mice and 7 control mice up to 18 weeks of age after tamoxifen injection at the age of 6 weeks. Seventy five percent of the cKO mice (9 out of 12) died by 18 weeks of age and the survived 3 cKO mice showed essentially the same tendency of serum values in phosphate, calcium, FGF23, 1,25(OH)_2_D and PTH as compared with control non-cKO mice of the same age. Control 18-week old non-cKO mice showed the same serum values in these minerals and hormones as compared with 9-week old non-cKO mice and survived without any growth retardation.

Control *Klotho-flox* heterozygous mice, *αKlotho*^*flox*/*+*^ *and Ndrg1-CreERT2;αKlotho*^*flox*/*+*^, did not show any phenotypic changes after tamoxifen treatment (Supplementary Table S2), suggesting that 50% reduction of *αKlotho* expression in proximal tubules does not affect the phenotypes.

### Parathyroid cells do not express Ndrg1-CreERT2 after tamoxifen treatment

**Figure 4:**
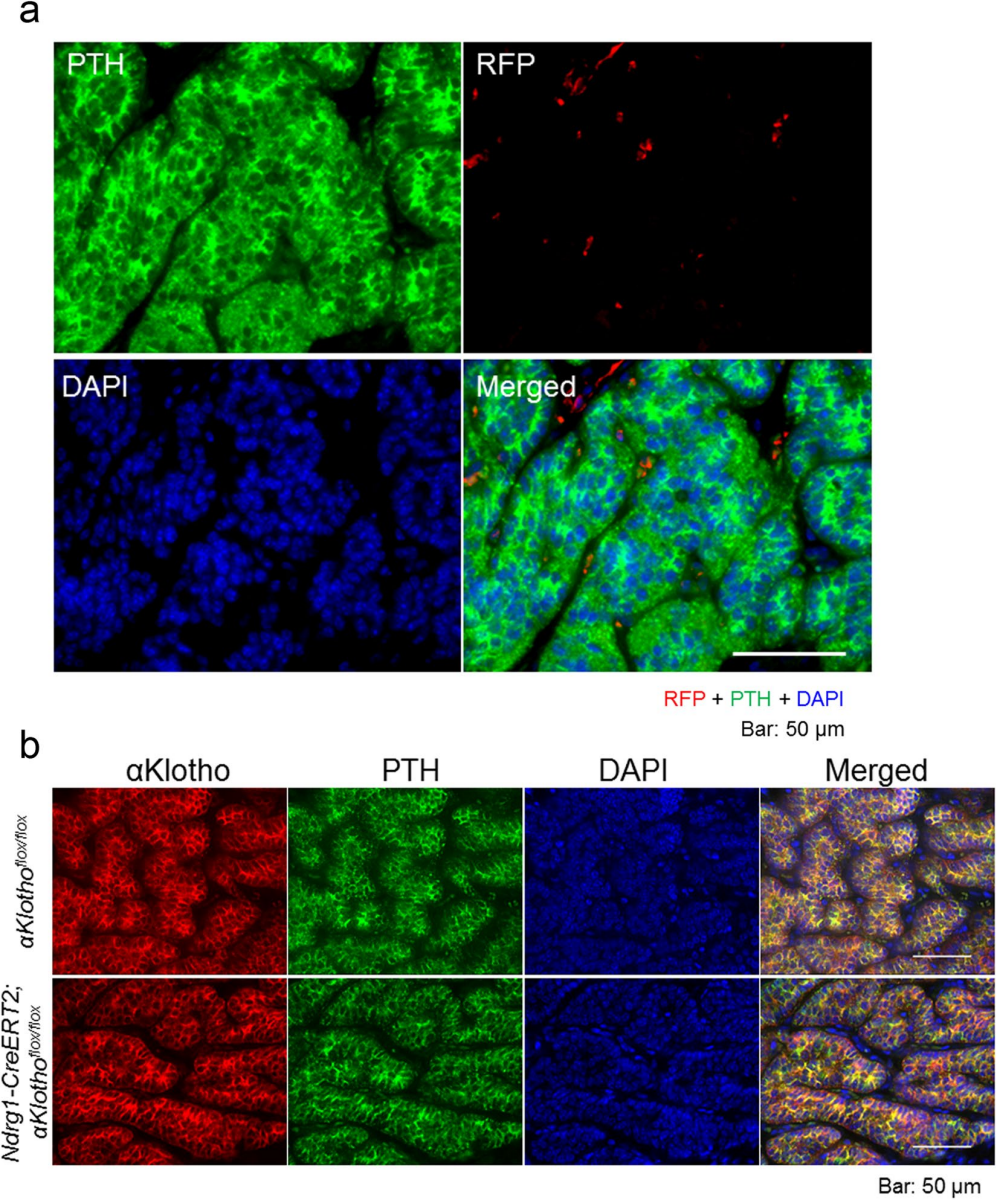
No expression of Ndrg1-CreERT2 in the parathyroid glands. (**a**) Cre expression study in the parathyroid glands of *Ndrg1-CreERT2;tdTomato* mice at 3 weeks after tamoxifen treatment. Parathyroid sections were immunostained for parathyroid hormone (PTH, green) and RFP (red), and counterstained for cell nuclei with DAPI (blue). Cre was not expressed in PTH-positive parathyroid cells. (**b**) αKlotho expression study in the parathyroid glands of *Ndrg1-CreERT2;αKlotho*^*flox*/*flox*^ mice and *αKlotho*^*flox*/*flox*^ control mice at 3 weeks after tamoxifen treatment. Parathyroid sections were immunostained for αKlotho (red) and PTH (green), and counterstained for cell nuclei with DAPI (blue). αKlotho was expressed equally in PTH-positive parathyroid cells of both mouse strains. Scale bars: 50 μm.

The parathyroid glands are another important organ that expresses αKlotho abundantly and influences systemic mineral metabolism. We reported that the increased levels of FGF23 in chronic renal disease stimulate αKlotho/FGFR signal transduction, enhancing the growth and hormone secretion of the parathyroid glands. Therefore, using *Ndrg1-Cr*e*ERT2;tdTomato*^*flox*/*flox*^ mice with tamoxifen injection, we examined the expression of RFP in parathyroid cells that stain positive for PTH. These cells did not express RFP at all, suggesting that *Ndrg1-CreERT2* is not expressed (Fig. 4a). Furthermore, αKlotho expression in the parathyroid glands was not affected by tamoxifen treatment, confirming the results of Fig. 4a (Fig. 4b).

### Proximal tubule-specific *Fgfr1–4* ablation induces ectopic calcification and loss of αKlotho expression in distal tubules, both of which are reversed by a vitamin D-deficient diet

**Figure 5:**
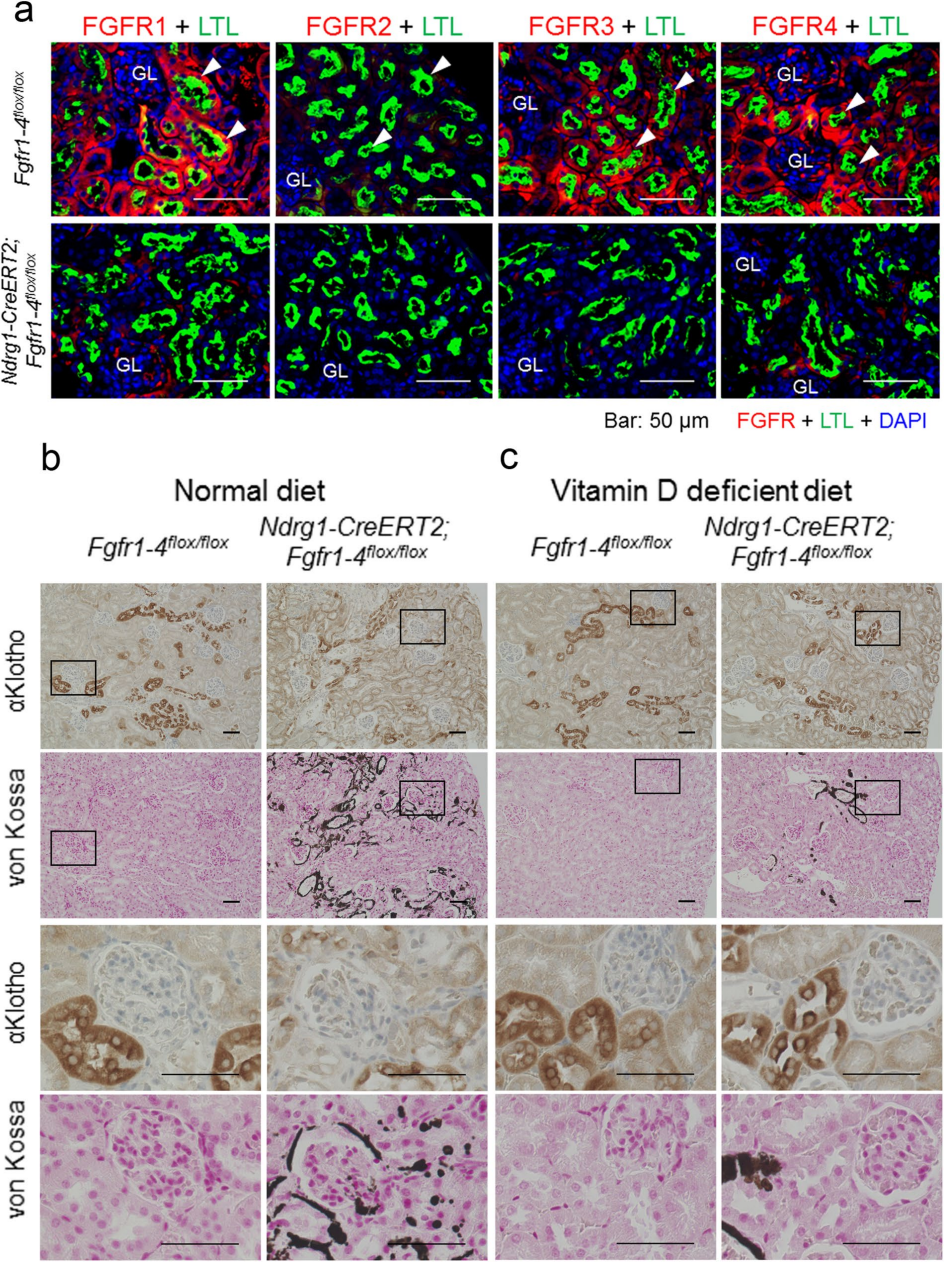
Proximal tubule-specific deletion of FGFR1*–*4 induces ectopic calcification and loss of αKlotho expression in distal tubules, both of which can be reversed by a vitamin D-deficient diet. (**a**) Indirect immunofluorescence staining studies for FGFR1, FGFR2, FGFR3, and FGFR4 in *Fgfr1–4*^*flox*/*flox*^ and *Ndrg1-CreERT2;Fgfr1–4*^*flox*/*flox*^ mice at 3 weeks after tamoxifen treatment. Kidney sections were immunostained for FGFR (red) with marking of proximal tubules with LTL (green) and counterstaining of cell nuclei with DAPI (blue). GL: glomerulus. Scale bars: 50 μm. (**b**) Proximal tubular ablation of FGFRs in mice maintained on a normal diet causes cortical ectopic calcification, disassembly of distal tubular cells, and loss of distal tubular αKlotho expression. Kidney serial sections were stained for αKlotho and calcium deposition using DAB-based immunostaining and von Kossa staining methods, respectively. Rectangular areas are magnified below in the two rows (αKlotho and von Kossa). Scale bars: 50 μm. (**c**) Reversal of cortical calcification, disassembly of distal tubular cells, and loss of distal tubular αKlotho expression in mice on a vitamin D-deficient diet. The experiments were carried out and shown as in (**b**). Kidney cortical surface is oriented to the right. The same experiments were repeated in 3 different littermates with and without the *Ndrg1-CreERT2* transgene under the C57BL/6 J *Fgfr1–4*^*flox*/*flox*^ background. Shown are representative micrographs. See also Supplementary Figure S3.

We found that proximal tubules expressed FGFR1, FGFR3, and FGFR4, but barely FGFR2 (Fig. 5a), which is consistent with the findings of others^26,27^. Tamoxifen-induced Cre expression in the *Ndrg1-CreERT2;Fgfr1–4*^*flox*/*flox*^ mice almost completely abrogated the expression of these FGFRs from proximal tubules (Fig. 5a). We also found ectopic calcification in the vascular walls and tubules in only the cortex (Supplementary Figure S4). The cortical area close to the surface, the corticomedullary boundary zone and the medullary area were free of calcification. FGFR expression was reduced in distal tubular cells as well (Fig. 5a), suggesting the damage to these cells caused by ectopic calcification. This is similar to the case in *αKlotho* cKO.

**Figure 6:**
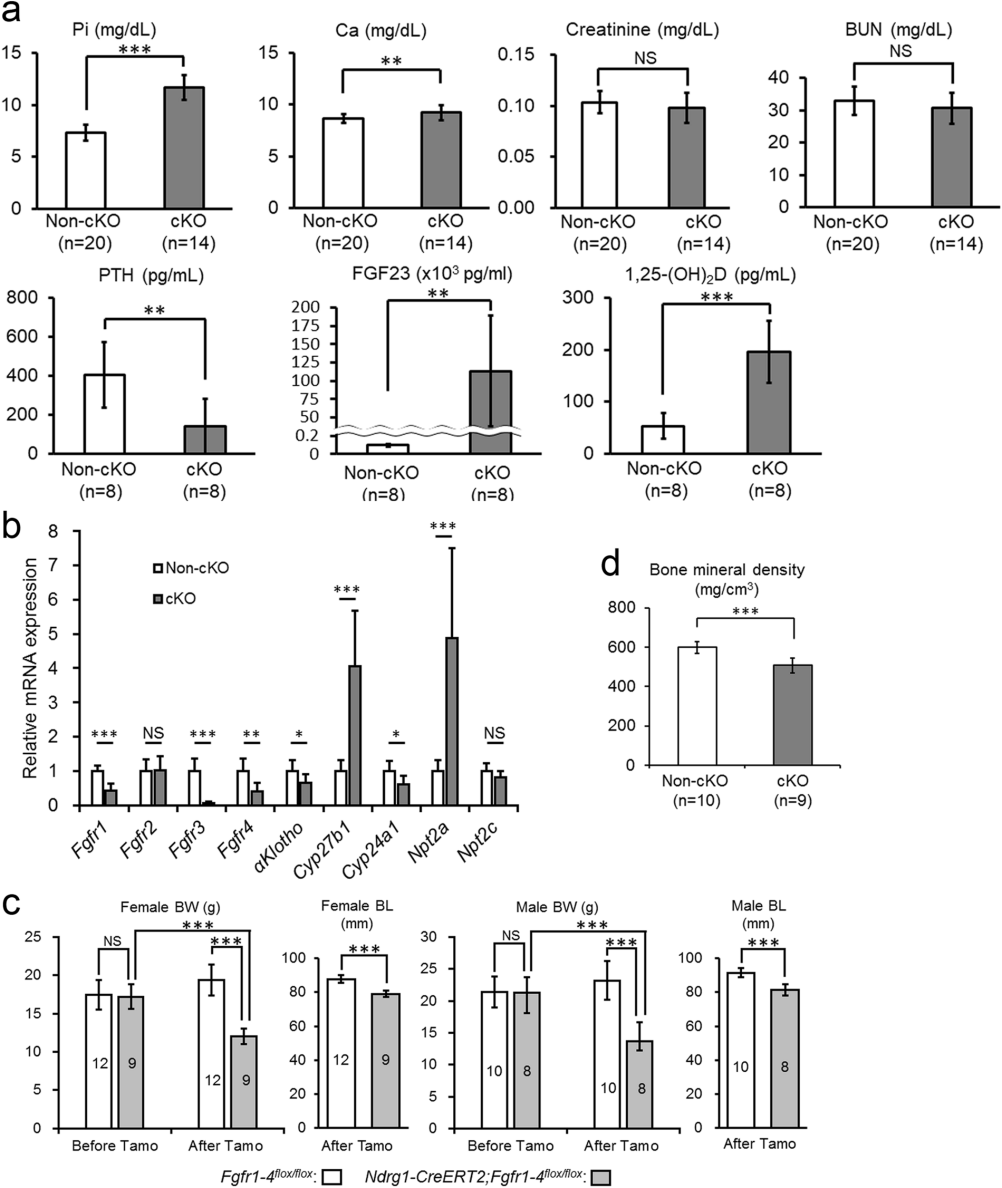
Proximal tubule-specific *Fgfr1–4* cKO reproduces the phenotypes of proximal tubule-specific *αKlotho* cKO. (**a**) Biochemical analysis of plasma inorganic phosphate (Pi), calcium (Ca), creatinine, blood urea nitrogen (BUN), parathyroid hormone (PTH), fibroblast growth factor 23 (FGF23), and 1,25-dihydroxyvitamin D (1,25(OH)_2_D) at 9 weeks of age at 3 weeks after the induction of Cre by tamoxifen treatment. These parameters were measured as described in the Methods section. Values are expressed as mean ± s.d. Number of samples (n) is described in the figure. (**b**) RT-qPCR of RNA extracted from the kidney. RT-qPCR was performed as described in the Methods section. Primers used for these studies are listed in the Supplementary Table S4. Values are expressed as mean ± s.d. of 8 samples for both non-cKO and cKO mice. (**c**) Mouse body size. Body weight (BW) was measured at 6 weeks of age before Cre induction and at 9 weeks of age at 3 weeks after Cre induction, whereas body length (BL) was measured only at 3 weeks after Cre induction. Data show a body size reduction in *Fgfr1-4* cKO mice as compared with non-cKO control mice. Values are expressed as mean ± s.d. Number of animals used in each group is indicated in each bar graph. (**d**) Bone analysis. Bone mineral density of the femurs was analysed with micro-CT as described in the Methods section. Values are expressed as mean ± s.d. of 10 samples. Scale bar: 5 cm. Statistical significance between 2 groups was examined by a two-tailed unpaired Student’s t test, whereas the significance among more than 3 groups was analysed by one-way ANOVA followed by Tukey’s multiple comparison test. NS, not significantly different; *P < 0.05; **P < 0.01; ***P < 0.001. See also Supplementary Table S3.

These distal cells showed marked reduction in αKlotho expression and morphological disassembly presumably caused by ectopic calcification, as shown in serial sections stained for αKlotho (DAB) and calcification (von Kossa) (Fig. 5b). αKlotho expression in proximal tubules remained unchanged in these mice (Fig. 5b). These findings except αKlotho expression in proximal tubules were very similar to those of mice with proximal tubule-specific *αKlotho* ablation. However, when fed a vitamin D-deficient diet as reported in systemic *αKlotho* KO mice^2^, these mice showed reduced ectopic calcification, nearly normal morphology and almost normal αKlotho expression in distal tubules compared with control mice (Fig. 5c), suggesting that cortical ectopic calcification is the main cause of morphological disassembly and loss of αKlotho expression in distal tubules. *Fgfr1-4* cKO and non-cKO mice fed a vitamin D-deficient diet showed low levels of 1,25(OH)_2_D (cKO vs. non-cKO mice: 7.7 ± 3.5 [n = 5] vs. 2.3 ± 1.0 pg/mL [n = 6]) as compared with the mice on a regular diet (cKO vs. non-cKO mice: 196.4 ± 59.6 pg/mL [n = 8] vs. 53.3 ± 24.5 pg/mL [n = 8]; see also Fig. 6a).

The same diet did not restore the distal tubular FGFR expression (data not shown due to similarity to Fig. 5a), suggesting a possibility that the distal tubular αKlotho and FGFR expression is downregulated differentially by high plasma levels of FGF23. FGF23 levels remained high in *Fgfr1–4* cKO mice compared with the control non-cKO mice even if they were fed a vitamin-D deficient diet (cKO vs. non-cKO: 112,115 ± 26,730 pg/mL [n = 4] vs. 102 ± 54 pg/mL [n = 2], P = 0.0036). The distal tubule FGFR expression might be more sensitive to high FGF23 levels than the αKlotho expression. Alternatively, it might be possible that the distal tubule FGFR expression is more sensitive to the tubular damage than the αKlotho expression. Vitamin D-deficient diet did not completely abolish the renal ectopic calcification.

### Proximal tubule-specific *Fgfr1–4* cKO changes the parameters of mineral metabolism and body size similarly to proximal tubule-specific *αKlotho* cKO

The effects of *Fgfr1–4* ablation on mineral metabolism were also very similar to those of *αKlotho* ablation from proximal tubules (Fig. 6a). *Ndrg1-CreERT2;Fgfr1–4*^*flox*/*flox*^ mice showed significant hyperphosphatemia (11.69 ± 1.19 mg/dL [n = 14] vs. 7.35 ± 0.76 mg/dL [n = 20], *P* = 2.6 × 10^−14^) and hypercalcemia (9.22 ± 0.71 mg/dL [n = 14] vs. 8.65 ± 0.44 mg/dL [n = 20], *P* = 0.0067) compared with *Fgfr1–4*^*flox*/*flox*^ control mice after tamoxifen treatment. The plasma values of creatinine (0.098 ± 0.015 mg/dL [n = 14] vs. 0.104 ± 0.011 mg/dL [n = 20], *P* = 0.208) and BUN (30.6 ± 4.9 mg/dL [n = 14] vs. 32.9 ± 4.3 mg/dL [n = 20], *P* = 0.163) were not different between both groups of mice. Humoral factors regulating mineral metabolism exhibited a decrease of plasma PTH values (138.5 ± 143.4 pg/mL [n = 8] vs. 404.1 ± 167.7 pg/mL [n = 8], *P* = 0.0043) and increases of both plasma FGF23 (113,279 ± 75,531 pg/mL [n = 8] vs. 121 ± 29 pg/mL [n = 8], *P* = 0.0039) and 1,25(OH)_2_D (196.4 ± 59.6 pg/mL [n = 8] vs. 53.3 ± 24.5 pg/mL [n = 8], *P* = 0.00013) values between both groups. These findings were also essentially the same as those of *Ndrg1-CreERT2;αKlotho*^*flox*/*flox*^ mice after tamoxifen treatment.

We quantified the expression of several mRNAs in the kidney using RT-qPCR with *Gapdh* as a reference gene to calibrate their expression (Fig. 6b). When compared with control animals (*Fgfr1–4*^*flox*/*flox*^) (n = 8) after tamoxifen injection, *Ndrg1-CreERT2;Fgfr1–4*^*flox*/*flox*^ (*Fgfr1-4* cKO) mice (n = 8) showed decreased mRNA expression of *Fgfr1* (by 57%, *P* = 2.7 × 10^−5^), *Fgfr3* (by 94%, *P* = 3.3 × 10^−6^), *Fgfr4* (by 59%, P = 0.0014), *αKlotho* (by 35%, *P* = 0.030), and *Cyp24a1* (by 39%, *P* = 0.015) and increased mRNA expression of *Cyp27b1* (4.1-fold, *P* = 0.00014) and *Npt2a* (4.9-fold, *P* = 0.00098). *Fgfr2* (*P* = 0.89) and *Npt2c* (*P* = 0.081) expression did not change significantly. *Fgfr2* is reportedly not expressed in proximal tubules^26,27^, supporting our finding. The other findings were completely consistent with our results in *αKlotho* cKO mice.

The gross appearance of these *Fgfr1–4*-ablated mice resembled that of *αKlotho*-ablated mice. *Fgfr1-4* cKO mice showed a significant reduction in body size after treatment with tamoxifen compared with control mice (Fig. 6c). Bone mineral density was also reduced in the cKO mice (Fig. 6d).

In the absence of tamoxifen treatment, *Ndrg1-CreERT2;Fgfr1–4*^*flox*/*flox*^ mice did not show any difference in the plasma parameters of mineral metabolism or in body size (Supplementary Table S3).

## Discussion

In this study, we found that most of the effects of FGF23 on mineral metabolism in mice are apparently mediated by αKlotho/FGFR co-receptors expressed in proximal tubules. The proximal tubule-specific KO of either *αKlotho* or *Fgfr1–4* reproduced almost all of the phenotypes of systemic *αKlotho* or *Fgf23* KO. The primary function of αKlotho is not attributed to its β-glucuronidase enzyme activity. The early signs of *αKlotho* or *Fgfr1–4* genetic deletion from proximal tubules were ectopic calcification of the vascular walls, glomeruli, and kidney tubules in the kidney cortex. This calcification was located only in the mid-cortex surrounding the glomeruli, where distal convoluted tubules are concentrated. Most proximal tubules appeared to be free of calcification.

**Figure 7:**
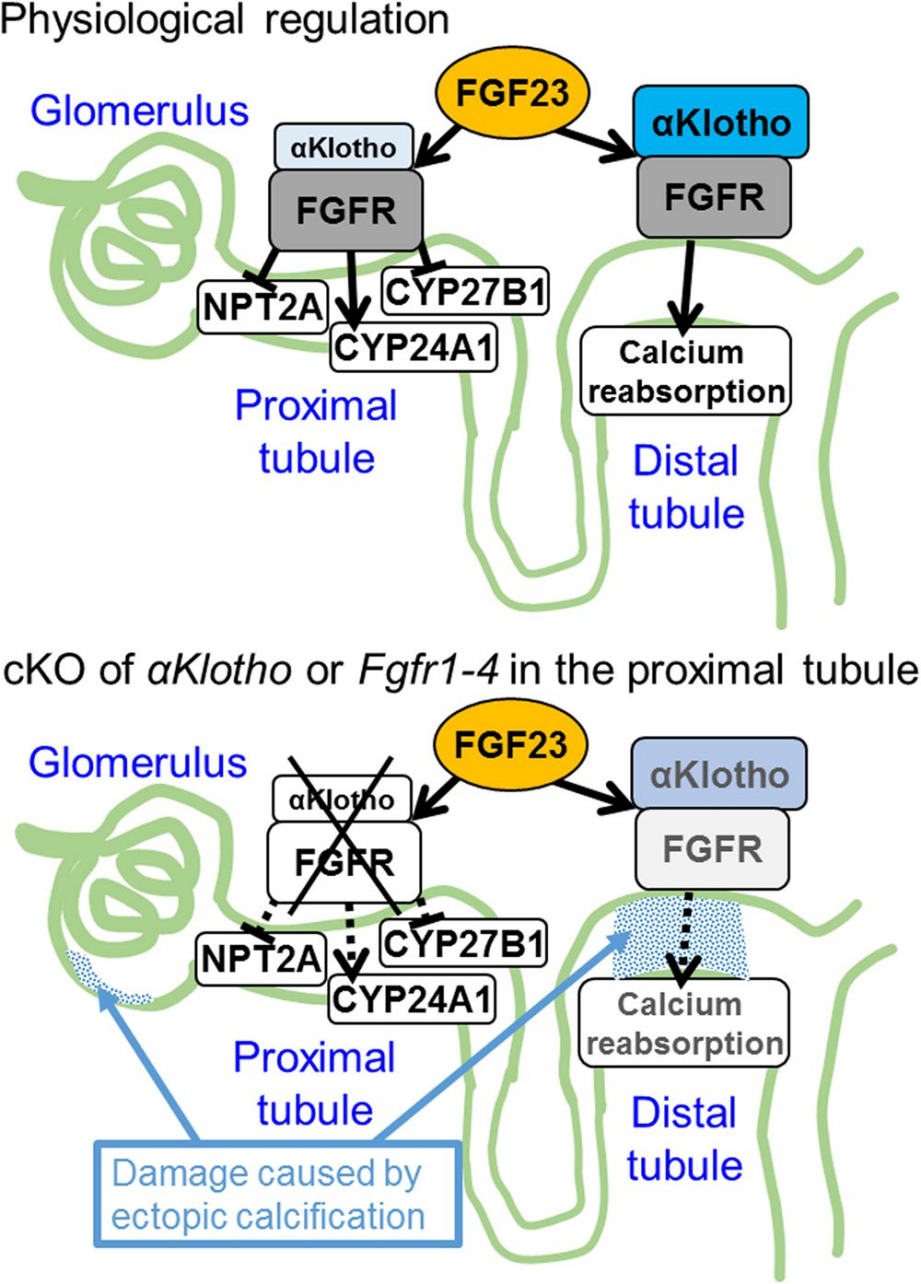
A scheme showing our results that FGF23 directly acts on proximal tubular cells. FGF23 plays critical roles in phosphate handling and vitamin D metabolism in the kidney. However, the effector cells of FGF23 have been controversial. αKlotho, a putative enzyme possessing β-glucuronidase activity and also a permissive co-receptor for FGF23 to bind to FGF receptors (FGFR), is most abundantly expressed in distal tubules whereas modestly in proximal tubules. FGFR are expressed in both proximal and distal tubules. The expression of a phosphate transporter, NPT2A, and vitamin D-metabolizing enzymes, CYP27B1 and CYP24A1, is confined to proximal tubules. The stimulatory effect of FGF23 on calcium reabsorption in distal tubules has been demonstrated by others^17,18^ and included in the scheme. Here, as shown in the bottom panel, we demonstrate, using Cre-loxP-mediated postnatal ablation of *αKlotho* or *Fgfr1-4* specifically from proximal tubular cells, that the primary function of FGF23 on phosphaturic effect and vitamin D metabolism is mediated through αKlotho/FGFR co-receptors expressed in proximal tubular cells. However, the resulting intra-renal ectopic calcification presumably damages the function of distal tubules, glomeruli and vasculature. T-shaped lines and arrowhead lines indicate inhibition and stimulation, respectively. Dotted lines indicate functional weakening or abrogation. Both αKlotho and FGFR are membrane-intercalated molecules.

αKlotho expression appears reduced in distal convoluted tubules in association with ectopic calcification in the kidney. This was similar in mice with proximal tubule-specific cKO of either *αKlotho* or *Fgfr1-4*. This ectopic calcification is reportedly one of the important phenotypes of systemic *αKlotho* KO mice^2^. Dietary restriction of vitamin D alleviates this calcification together with most of the other phenotypes of these mice^2^. Our own study using a vitamin D-deficient diet in *αKlotho or Fgfr1–4* cKO mice replicated these results although ectopic calcification was not abolished completely in *Fgfr1-4* cKO mice. In these mice, αKlotho expression remained normal in distal tubules, suggesting that the reduced distal tubular αKlotho expression in cKO mice with a normal diet is due to tubular damage caused by ectopic calcification and that the proximal tubule is the primary site at which FGF23 regulates phosphate and vitamin D metabolism. Needless to say, cortical calcification accompanying the reduced expression of αKlotho in distal tubules have secondary effects on mineral metabolism. Distal tubules are known to be the site of FGF23-mediated calcium reabsorption^17,18^. Current knowledge of FGF23 functions in kidney tubules is schematically shown in Fig. 7.

Our results are different from those of two previous studies^15,19^ that were conducted using different strains of *Cre* and *αKlotho*^*flox*/*flox*^ mice. These differences might be explained by recombination efficiency caused by the following three factors: cell-type specificity of Cre expression, Cre expression efficiency in specific cell types, and recombination feasibility, which is defined by the structure of the lox P insertion site in the genome. The first study intended to study the function of αKlotho in distal tubules using *Ksp-cadherin-Cre* transgenic mice^15^. The problems here were probably both the cell-type specificity of Cre expression and the Cre expression efficiency in a specific cell type^16^.

The second study examined the function of αKlotho in proximal tubules using three different inducible Cre expression mouse strains (*PEPCK-Cre*, *Kap-Cre*, and *Slc34a1-Cre*)^19^ mated with *αKlotho*^*flox*/*flox*^ mice. These mice showed mild or no hyperphosphatemia under basal dietary conditions. The effects on 1,25(OH)_2_D and FGF23 varied among the *Cre-αKlotho flox* lines, but were modest overall and not statistically significant. *PEPCK-Cre* and *Kap-Cre* mice showed only 70% and 60% recombination efficiency, respectively, probably as a result of low Cre expression efficiency, though other causes cannot be excluded. The *Slc34a1-Cre* mice, however, were shown to have good recombination efficiency in proximal tubules. In *Slc34a1-Cre* mice, tamoxifen-inducible Cre expression is under the control of the *Npt2a* gene (also called *Slc34a1*). *Slc34a1-Cre;αKlotho*^*flox*/*flox*^ mice showed weak hyperphosphatemia consistent with αKlotho deletion and significantly increased *Npt2a* mRNA expression. However, they did not show any increase in FGF23 or 1,25(OH)_2_D serum levels. The expression of *Cyp27b1* and *Cyp24a1* mRNA was not affected. These findings suggest that NPT2A and vitamin D-metabolizing enzymes might be expressed differentially in individual cells of the proximal tubule. Incidentally, FGF23 appears to decrease NPT2A expression via FGFR1^26^ and regulate serum 1,25(OH)_2_D via FGFR3 and FGFR4^28^. These findings might imply the heterogeneity of proximal tubular cells.

We would also like to point out that the *αKlotho*^*flox*/*flox*^ mouse strain used for these previous studies was different from ours^23^. Both *αKlotho*^*flox*/*flox*^ mouse strains were used independently to evaluate the function of αKlotho in the parathyroid glands^23,29^. Upon ablation of *αKlotho* specifically from the parathyroid glands, we showed the clear suppression of both parathyroid cell growth and PTH secretion in mice with chronic kidney disease^23^. In contrast to our results, they did not find any effect of *αKlotho* ablation on parathyroid function in chronic kidney disease mice^29^. These findings suggest that the two *αKlotho*^*flox*/*flox*^ mouse strains might have different recombination feasibility for Cre recombinase.

The specificity of Ndrg1-CreERT2 expression in mice has been examined extensively^24^. We have also examined it in the parathyroid glands, which express αKlotho abundantly and secrete one of the most important hormones (PTH) influencing mineral metabolism, showing no expression of Cre following tamoxifen injection. Bone is another tissue that might have significant effects on mineral metabolism. However, a recent report clearly shows that *αKlotho* KO specifically from osteocytes does not affect the plasma values of any biochemical and humoral factors^30^, suggesting that αKlotho in osteocytes, even if deleted, does not cause the phenotypes presented here.

In summary, we found that αKlotho expressed in proximal tubules plays a central role in FGF23-mediated mineral metabolism. One of the earliest changes in the kidney of *αKlotho* or *Fgfr1-4* proximal-tubule cKO mice is ectopic calcification in the mid-cortex, which damages the function of glomeruli, vasculature, and distal tubules. Eventually, αKlotho expression is also reduced in distal tubules. We conclude that FGF23 primarily affects the metabolism of phosphate and vitamin D through αKlotho/FGFR co-receptors expressed in proximal tubules.

## Methods

### Animal experiments

This study was carried out in strict accordance with the recommendations in the Guide for the Care and Use of Laboratory Animals of the National Institutes of Health. The protocol was approved by the Committee on the Ethics of Animal Experiments of the Wakayama Medical University (Permit Number: 557). All surgery and sacrifice were performed under sodium pentobarbital anesthesia, and all efforts were made to minimize animal suffering.

### Generation of proximal tubule-specific *αKlotho* and *Fgfr1–4* KO mice

We generated *αKlotho*^*flox*/*flox*^ and *Fgfr1–4*^*flox*/*flox*^ mice as reported^23^. The sources of the mice were as follows: *Fgfr1*^*flox*^ mouse^31^ from Dr. Deng of NIDDK (Bethesda, MD); *Fgfr2*^*flox*^ mouse from Dr. Ornitz^32^ of the Washington University Medical School (St. Louis, MO); *Fgfr3*^*flox*^ mouse^33^ from Dr. Chen of the Third Military Medical University (Chongqing, China); *αKlotho*^*flox*^ and *Fgfr4*^*flox*^ mice were generated in our laboratory^23^. These mouse strains were mated with *Ndrg1-CreERT2* transgenic mice, which express CreERT2 in proximal tubules in a tamoxifen-dependent manner^24^. All mice were kept on a genetic background of C57BL/6 J. Mouse genotypes were determined as described previously^23,24^. *Ndrg1-CreERT2;αKlotho*^*flox*/*flox*^ and *αKlotho*^*flox*/*flox*^ mice were generated by mating *Ndrg1-CreERT2;αKlotho*^*flox*/*flox*^ mice with *αKlotho*^*flox*/*flox*^ mice; *Ndrg1-CreERT2;Fgfr1-4*^*flox*/*flox*^ and *Fgfr1-4*^*flox*/*flox*^ mice were generated by mating *Ndrg1-CreERT2;Fgfr1-4*^*flox*/*flox*^ mice with *Fgfr1-4*^*flox*/*flox*^ mice. All of these mice were used without any inclusion/exclusion criteria until sample sizes became the estimated numbers to fulfill the statistical criteria (also see the **Statistical analysis** subsection). No randomization was used. No blinding method was involved since their phenotypes were obvious at the time of sacrifice. Histological and biochemical comparisons were carried out using littermates from at least 3 different litters.

We administered tamoxifen (3 mg/20 g body weight; Sigma-Aldrich, St. Louis, MO) in sunflower oil to the flox mice, both with and without the *Ndrg1-CreERT2* transgene, by intraperitoneal injection for 5 consecutive days at 6 weeks of age and sacrificed them for biochemical and histological analysis and measurements of body size and bone mineral density at 9 weeks of age. For some histological analysis of the kidney, we injected tamoxifen at 5-8 weeks of age for 5 consecutive days and sacrificed mice at 1, 2 and 3 weeks after the initial injection. The age of mice did not affect the histological results but the periods after tamoxifen injection did. The mice used in this study were fed either a regular diet containing 1.07% calcium, 0.83% inorganic phosphate, and 137 IU/100 g vitamin D3 (MF; Oriental Yeast Co., Ltd, Suita, Japan) or a vitamin D-deficient diet containing 0.6% calcium, 0.4% inorganic phosphate, and no vitamin D3 (AIN-93G-based diet; Oriental Yeast Co., Ltd.). The vitamin D-deficient diet was given to the female mice after confirming their mating according to a vaginal plug check and the same diet was administered to their offspring until sacrifice.

### Histology methods and reagents

Kidneys and thyro-parathyroid glands were fixed overnight in 4% paraformaldehyde at 4 °C. They were dehydrated in ethanol and embedded in paraffin wax. Paraffin-embedded paraformaldehyde-fixed kidneys and thyro-parathyroid glands were sectioned at 4-μm and 6-μm thickness, respectively. Then, the sections were immunostained using the standard indirect immunofluorescence technique. The primary antibodies used in the study were for RFP (ab62341; Abcam, Cambridge, UK), calbindinD28K (SAB4200543; Sigma-Aldrich), aquaporin 2 (#178612; Calbiochem-Novabiochem Corp., San Diego, CA), αKlotho (Catalog #KO603; Clone #KM2076; Transgenic Inc., Ltd., Kobe, Japan), FGFR1 (sc-121; Santa Cruz Biotechnology, Inc., Santa Cruz, CA), FGFR2 (sc-122; Santa Cruz Biotechnology, Inc.), FGFR3 (sc-123; Santa Cruz Biotechnology), FGFR4 (sc-9006; Santa Cruz Biotechnology), and parathyroid hormone (#7170-6216; clone BGN/1F8; Bio-Rad Laboratories, Tokyo, Japan) with DAPI (Thermo Fisher Scientific K.K., Waltham, MA) co-staining for cell nuclei or LTL-FITC (FL-1321; Vector Laboratories, Inc., Burlingame, CA) co-staining for proximal tubules. The secondary antibodies used for indirect immunofluorescence staining were as follows: Alexa Fluor 647-, 568-, or 488-conjugated anti-rabbit, -mouse, or -goat IgG produced in goat or donkey (Molecular Probes, Eugene, OR).

For immunohistochemical studies, the sections were incubated with an anti-αKlotho antibody (#KO603; Transgenic Inc., Ltd.), and detected by staining with 3,3′-diaminobenzidine (DAB) substrate (Nakalai Chemicals Ltd., Kyoto, Japan) and counterstaining with hematoxylin using the standard technique. A test experiment was carried out to show specificity of this anti-αKlotho antibody (Supplementary Fig S5**)**. To detect calcification, the sections were stained with von Kossa staining according to the standard histologic protocol. Nuclear fast red was used for counterstaining nuclei. HE staining was carried out using the standard technique.

### RT-qPCR

To quantify mRNA expression levels in the kidney, cDNA was synthesized with a High-Capacity cDNA Reverse Transcription Kit (Product #4368814; Thermo Fisher Scientific, Inc.) and qPCR was performed and analysed with an iCycler iQ Real-Time PCR Detection System with 1 × IQ SYBR Green Supermix (Catalog #170-8880; Bio-Rad Laboratories, Tokyo, Japan) and 5-μM primers. The primer sequences for each gene are shown in Supplementary Table S4. The PCR conditions were 95 °C for 3 min followed by 40 cycles of 95 °C for 10 s and 60 °C for 30 s. Relative mRNA expression levels were calculated using the 2^−ΔΔCT^ method^34^ to normalize target gene mRNA to *Gapdh* as reported previously^35^.

### Serum biochemistry analysis

Blood was drawn into a heparinized syringe, and plasma was separated by centrifugation at 4,000 rpm for 15 min. The plasma concentrations of calcium, phosphate, BUN, and creatinine were measured by an autoanalyser using an Aqua-auto Kainos Calcium Kit (Kainos Laboratories, Inc., Tokyo, Japan), a Determiner L IP II Kit (Kyowa Medex Co., Ltd., Tokyo, Japan), Aqua-auto Kainos UN-II Kit (Kainos Laboratories, Inc.), and an Aqua-auto Kainos Creatinine Kit (Kainos Laboratories, Inc.), respectively. The plasma levels of FGF23, 1,25(OH)_2_D, and PTH were quantified using an FGF23 ELISA Kit (Kainos Laboratories, Inc.), a 1,25(OH) -Vitamin D-RIA-CT Kit (DIAsource ImmunoAssays, Louvain-la-Neuve, Belgium), and a Mouse PTH(1–84) ELISA Kit (Immunotopics, Inc., San Clemente, CA), respectively.

### Micro-CT analysis of the femur

Femurs were resected after sacrifice at 9 weeks of age. Muscles, fat, and ligaments were removed as much as possible from the bone, and the bones were kept in 70% ethanol until the time of measurement (for up to 1 month). Bone mineral density of the entire right femur was measured using a LaTheta LCT-200 micro-CT analyser (Hitachi-Aloka Medical Ltd., Tokyo, Japan). Tube voltage was set at 50 kV and current was constant at 0.5 mA. Bones were scanned in a 24-mm-wide specimen holder with a pixel size of 48 × 48 µm, slice size of 48 µm, and pitch size of 48 µm. For all scans, the same number of views (n = 1592) was used, which represents the number of data collected during a single 360° rotation.

### Statistical analysis

All experiments were repeated at least three times. All data are presented as mean ± s.d. The statistical significance between two unpaired groups was analysed by a two-tailed Student’s t test, whereas the significance among more than 3 groups was examined by one-way ANOVA followed by the Tukey’s multiple comparison test. Data distribution normality was examined by the Kolmogorov–Smirnov test. Statistical analyses were performed using Excel 2013 (Microsoft) or GraphPad Prism6.0. Optimum sample size was calculated using StatsDirect version 3.1.11 software. NS, not significantly different; *P < 0.05; **P < 0.01; ***P < 0.001.

### Data availability

The datasets generated and/or analysed during the current study are available from the corresponding author on reasonable request.

## Electronic supplementary material

Supplementary Information
