# Supplementary material for: Central role of the proximal tubular αKlotho/FGF receptor complex in FGF23-regulated phosphate and vitamin D metabolism
Source: Sci Rep. 2018 May 2;8:6917. doi: 10.1038/s41598-018-25087-3 (PMC5932018; doi:10.1038/s41598-018-25087-3)
Supplement: Supplementary file 1 — Supplementary Information [file 41598_2018_25087_MOESM1_ESM.pdf]

Supplementary information

Central role of the proximal tubular αKlotho/FGF receptor complex in FGF23-regulated phosphate and vitamin D metabolism

Ai Takeshita[1], Kazuki Kawakami[1], Kenryo Furushima[1], Masayasu Miyajima[2], and Kazushige Sakaguchi[1]*

[1]Department of Molecular Cell Biology and Molecular Medicine, Institute of Advanced Medicine, Wakayama Medical University, 811-1 Kimiidera, Wakayama 641-8509, Japan

[2] Laboratory Animal Center, Wakayama Medical University, 811-1 Kimiidera, Wakayama 641-8509, Japan

*Correspondence and material requests should be addressed to K.S. (ksaka@wakayama-med.ac.jp)

Table of contents:

Supplementary Figure S1
Supplementary Figure S2
Supplementary Figure S3
Supplementary Figure S4
Supplementary Figure S5
Supplementary Table S1
Supplementary Table S2
Supplementary Table S3
Supplementary Table S4

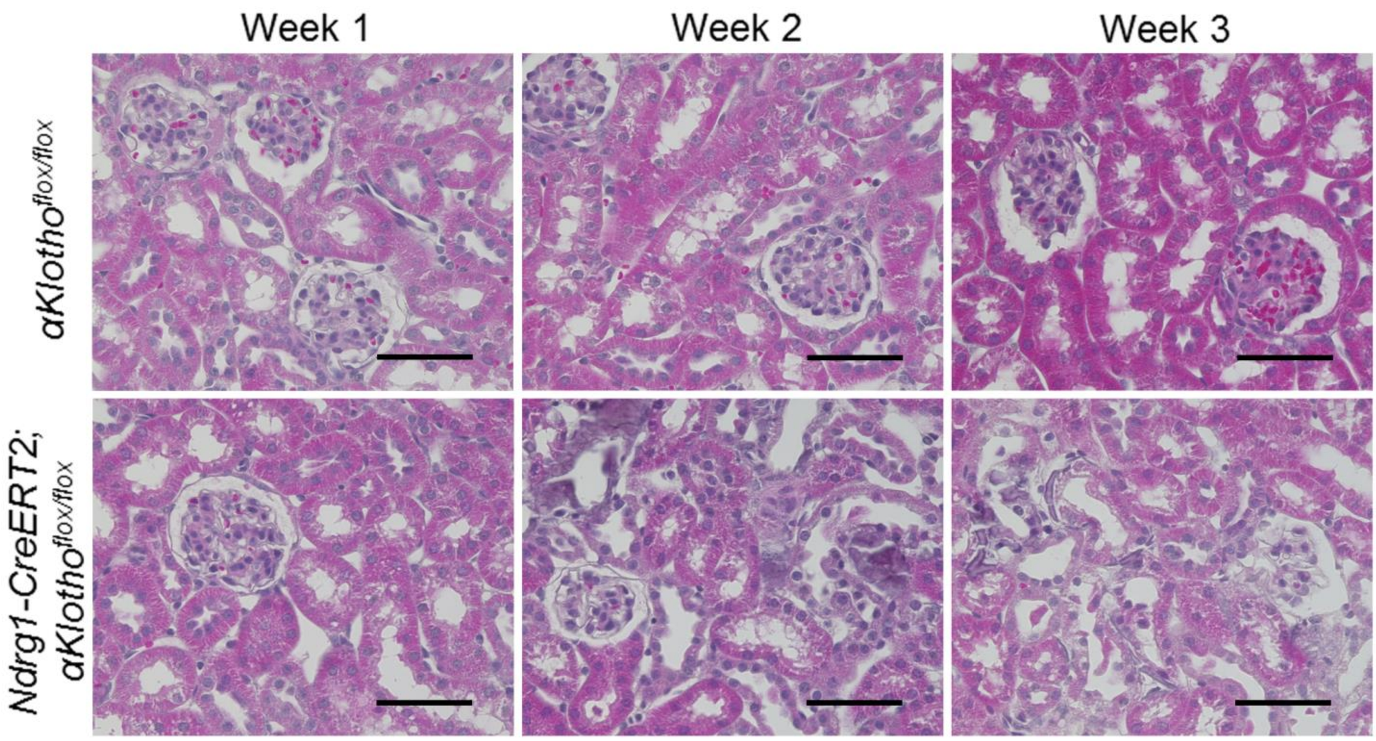

Supplementary Figure S1. HE staining of the kidney sections from *αKlotho*$^{flox/flox}$ and *Ndrg1-CreERT2;αKlotho*$^{flox/flox}$ mice at 1, 2, and 3 weeks after tamoxifen treatment. The structure of renal tubules remained apparently normal at 1 week after tamoxifen treatment, whereas the treatment caused morphological disassembly of kidney tubules and glomeruli after 2 and 3 weeks. Scale bars: 50 μm.

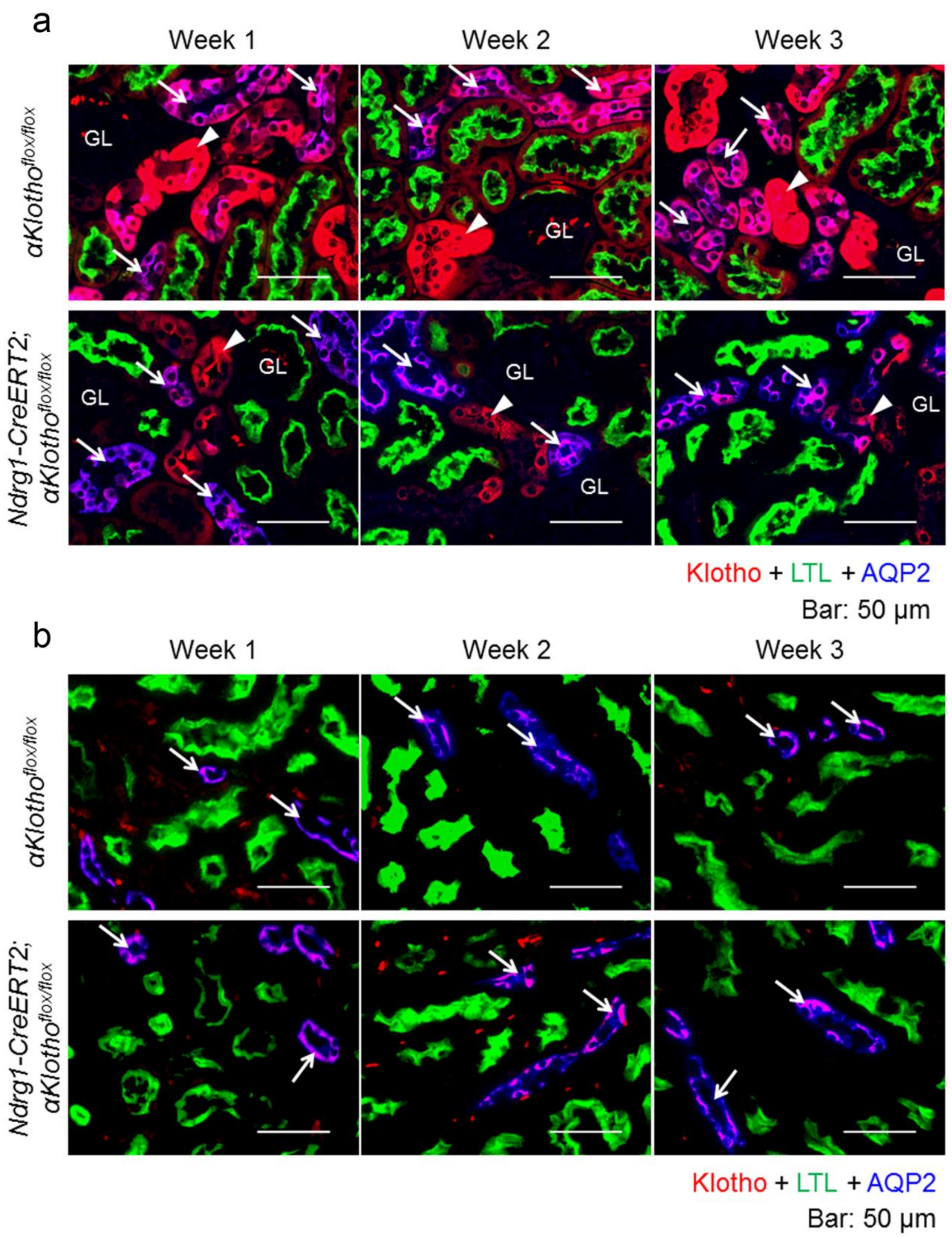

Supplementary Figure S2. Expression of αKlotho in the kidney cortex (a) and corticomedullary boundary zone (b) of *αKlotho*$^{flox/flox}$ and *Ndrg1-CreERT2;αKlotho*$^{flox/flox}$ mice at 1, 2, and 3 weeks after tamoxifen treatment. Kidney sections were co-stained for αKlotho and aquaporin 2 (AQP2; a marker for collecting ducts shown in blue) with affinity marking of renal tubules with lotus tetragonolobus lectin (LTL, green). LTL+ cells in the cortex are mostly proximal tubular cells, whereas most LTL+ cells in the boundary zone represent cells of descending limb of Henle's loop, which appears negative for αKlotho. Arrowheads in (a) indicate distal convoluted tubules expressing αKlotho. In both portions of the nephron, Ndrg1-CreERT2 induction did not change αKlotho expression in collecting ducts (arrows). GL: glomerulus. Scale bars: 50 μm.

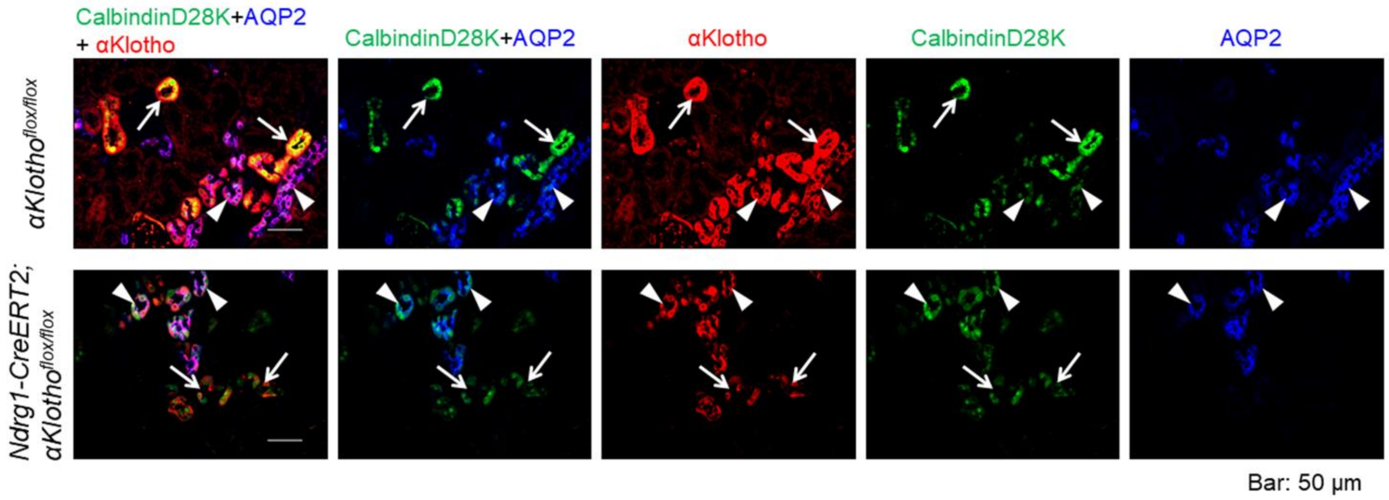

Supplementary Figure S3. Expression of αKlotho in distal convoluted tubules and connecting tubules in *αKlotho*$^{flox/flox}$ and *Ndrg1-CreERT2;αKlotho*$^{flox/flox}$ mice at 3 weeks after tamoxifen treatment. Kidney sections were co-stained for αKlotho (red), calbindinD28K (a marker for distal convoluted tubules shown in green) and aquaporin 2 (AQP2; a marker for collecting ducts shown in blue). Arrows indicate distal convoluted tubules labeled only with calbindinD28K, whereas arrowheads indicate connecting tubules labeled with both calbindinD28K and AQP2. Tamoxifen treatment disassembled the cells of distal convoluted tubules and decreased their αKlotho expression, but barely altered the structure or αKlotho expression of connecting tubules.

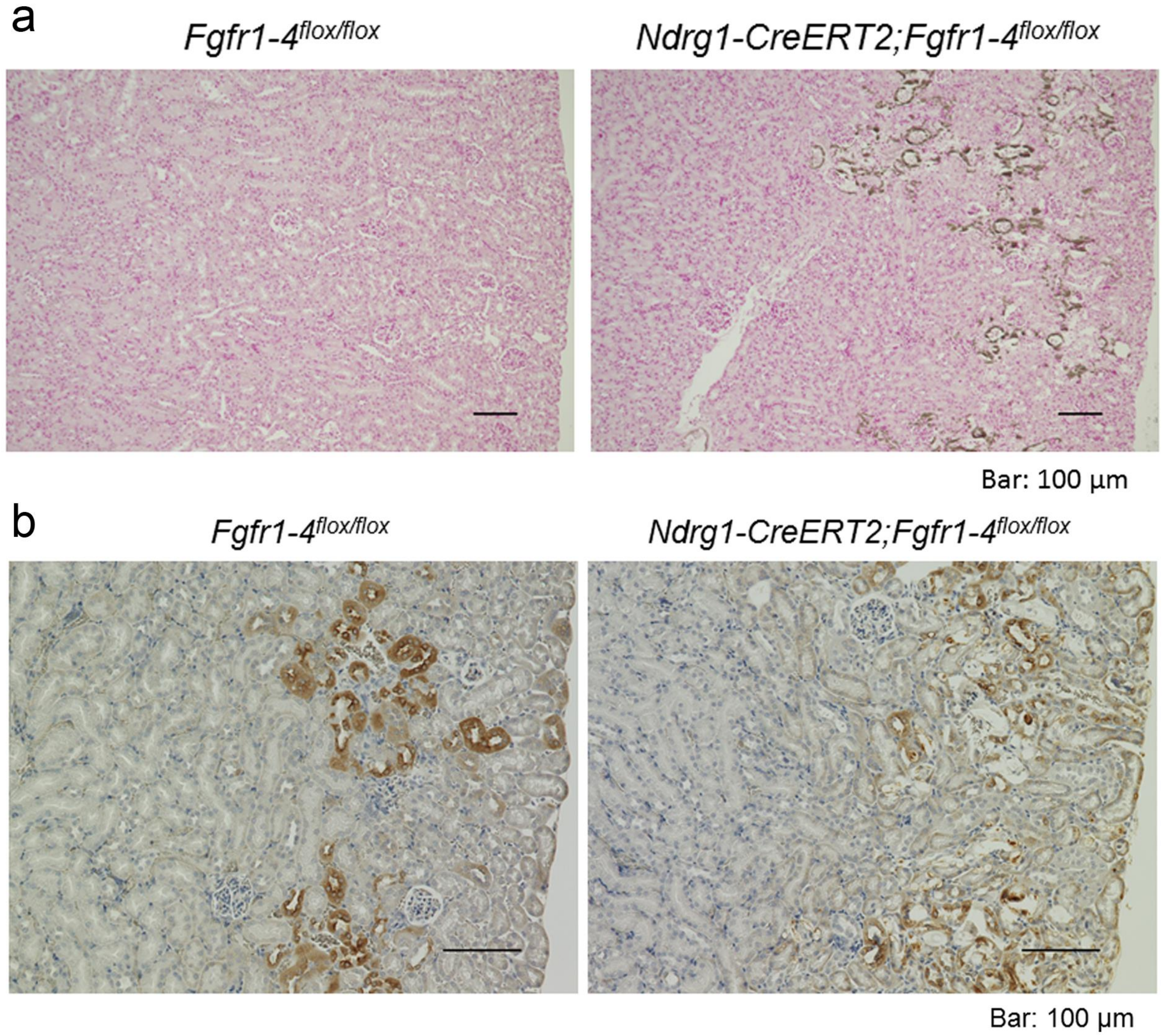

Supplementary Figure S4. Detection of ectopic calcification and αKlotho expression in the kidney sections of *Fgfr1–4*$^{flox/flox}$ and *Ndrg1-CreERT2;Fgfr1–4*$^{flox/flox}$ mice 3 weeks after tamoxifen treatment. a. Calcified tissue (dark brown) in the kidney. Kidney sections were stained for calcified tissue using the von Kossa method with counterstaining by nuclear fast red. A large amount of calcified tissue was formed in only the cortex area of *Ndrg1-CreERT2;Fgfr1–4*$^{flox/flox}$ mice after tamoxifen treatment. Kidney cortical surface is oriented to the right. b. DAB staining for αKlotho (dark brown) in the kidney cortex of *Fgfr1–4*$^{flox/flox}$ and *Ndrg1-CreERT2;Fgfr1–4*$^{flox/flox}$ mice after tamoxifen treatment. Kidney sections were immunostained for αKlotho with counterstaining by hematoxylin. In control *Fgfr1–4*$^{flox/flox}$ mice, distal convoluted tubules stained strongly positive, whereas proximal tubules stained mildly positive. In *Ndrg1-CreERT2;Fgfr1–4*$^{flox/flox}$ mice, the kidney cortex had a number of non-stained areas with decreased staining for αKlotho in distal convoluted tubules. αKlotho expression remained unchanged in proximal tubules. Kidney cortical surface is oriented to the right. The same experiments were repeated in 3 different litters using a pair of littermates with and without *Ndrg1-CreERT2 transgene* under the C57BL/6J *Fgfr1-4*$^{flox/flox}$ background. Shown are the representative micrographs.

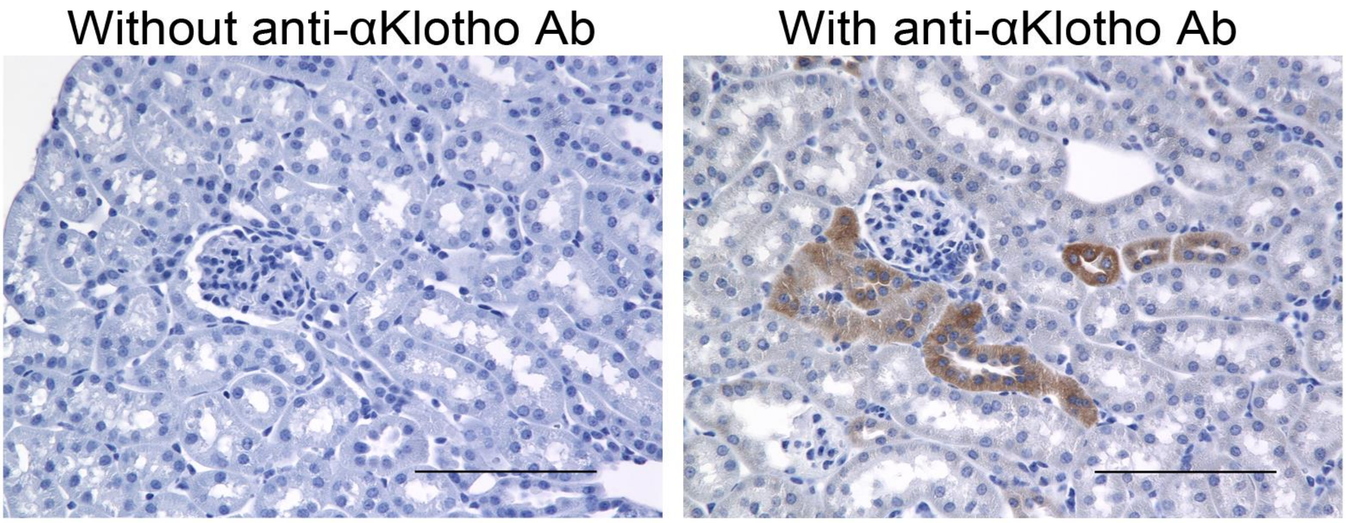

Supplementary Figure S5. Specificity of anti- α Klotho antibody used in our study. Kidney sections were incubated with or without an anti-αKlotho antibody (#KO603; Transgenic Inc., Ltd.), and detected by staining with 3,3′-diaminobenzidine (DAB) substrate (Nakalai Chemicals Ltd., Kyoto, Japan) and counterstaining with hematoxylin using the standard technique. Background without the primary anti-αKlotho antibody does not show any brown coloring. Distal tubules express abundant αKlotho and are stained in dark brown while proximal tubules express modest amount of αKlotho and are stained in light brown. Scale bars: 100 μm.

Supplementary Table S1. Plasma biochemistry and micro-CT analysis of α*Klotho* flox mice without tamoxifen treatment.

Plasma biochemistry

	α*Klotho*$^{flox/flox}$	*Ndrg1-CreERT2; αKlotho*$^{flox/flox}$	P value
Pi (mg/dL)	6.33 ± 1.10 (n = 10)	6.55 ± 1.29 (n = 11)	0.68
Ca (mg/dL)	8.76 ± 0.25 (n = 10)	8.35 ± 0.70 (n = 11)	0.10
PTH (pg/mL)	234.84 ± 187.69 (n = 8)	167.51 ± 73.74 (n = 8)	0.36
FGF23 (pg/mL)	200.55 ± 49.55 (n = 8)	205.92 ± 56.71 (n = 6)	0.85
1,25(OH)$_2$D (pg/mL)	39.22 ± 24.48 (n = 4)	38.57 ± 19.61 (n = 4)	0.97

Micro-CT analysis of the femur

	α*Klotho*$^{flox/flox}$	*Ndrg1-CreERT2;αKlotho*$^{flox/flox}$	P value
BMD (mg/cm^3)	549.3 ± 26.3 (n = 10)	561.9 ± 43.2 (n = 10)	0.44

Pi, inorganic phosphate; Ca, calcium; PTH, parathyroid hormone; FGF23, fibroblast growth factor 23; 1,25(OH)$_2$D, 1,25-dihydroxyvitamin D; BMD, bone mineral density; CT, computed tomography.

Supplementary Table S2. Plasma biochemistry, body size, and micro-CT analysis of heterozygous α*Klotho* flox mice with tamoxifen treatment.

Plasma biochemistry

	αKlotho$^{flox/+}$	*Ndrg1-CreERT2; αKlotho*$^{flox/+}$	P value
Pi (mg/dL)	6.62 ± 0.87 (n = 11)	6.75 ± 0.89 (n = 13)	0.73
Ca (mg/dL)	8.56 ± 0.23 (n = 11)	8.59 ± 0.55 (n = 13)	0.87
PTH (pg/mL)	221.30 ± 139.59 (n = 8)	174.57 ± 98.09 (n = 8)	0.45
FGF23 (pg/mL)	133.90 ± 45.67 (n = 8)	138.80 ± 51.01 (n = 8)	0.84
1,25$(OH)_2$D (pg/mL)	64.28 ± 23.84 (n = 4)	46.54 ± 8.69 (n = 4)	0.21

Body size

	αKlotho$^{flox/+}$	*Ndrg1-CreERT2;αKlotho*$^{flox/+}$	P value
Female BW before Tamo	18.30 ± 0.96 (n=6)	17.32 ± 0.53 (n=6)	0.052
Female BW (g) after Tamo	20.32 ± 1.50 (n = 6)	19.50 ± 0.97 (n = 8)	0.24
Female length (mm) after Tamo	90.17 ± 1.47 (n = 6)	88.63 ± 1.60 (n = 8)	0.090
Male BW (g) before Tamo	21.56 ± 0.76 (n = 5)	20.95 ± 1.44 (n = 6)	0.42
Male BW (g) after Tamo	24.38 ± 1.14 (n=5)	23.65 ± 1.65 (n= 6)	0.42
Male length (mm) after Tamo	24.38 ± 1.14 (n = 5)	23.65 ± 1.65 (n = 6)	0.43

Micro-CT analysis of the femur

	αKlotho$^{flox/+}$	*Ndrg1-CreERT2; αKlotho*$^{flox/+}$	P value
BMD (mg/cm^3)	625.0 ± 20.6 (n = 7)	620.5 ± 15.7 (n = 7)	0.66

Pi, inorganic phosphate; Ca, calcium; PTH, parathyroid hormone; FGF23, fibroblast growth factor 23; 1,25$(OH)_2$D, 1,25-dihydroxyvitamin D; BW, body weight; length, nose-to-anus length; BMD, bone mineral density; CT, computed tomography; Tamo, tamoxifen treatment.

Supplementary Table S3. Plasma biochemistry, body size, and micro-CT analysis of *Fgfr1–4* flox mice without tamoxifen treatment.

Plasma biochemistry

	Fgfr1–4$^{flox/flox}$	*Ndrg1-CreERT2;Fgfr1–4*$^{flox/flox}$	P value
Pi (mg/dL)	7.83 ± 1.46 (n = 13)	7.79 ± 1.92 (n = 13)	0.95
Ca (mg/dL)	9.22 ± 0.82 (n = 13)	8.74 ± 0.62 (n = 13)	0.10
PTH (pg/mL)	607.31 ± 454.44 (n = 6)	481.15 ± 114.84 (n = 8)	0.53
FGF23 (pg/mL)	157.79 ± 55.79 (n = 8)	144.73 ± 41.96 (n = 8)	0.61
1,25(OH)$_2$D (pg/mL)	51.38 ± 21.91 (n = 4)	47.27 ± 6.16 (n = 4)	0.73

Body size

	Fgfr1–4$^{flox/flox}$	*Ndrg1-CreERT2;Fgfr1–4*$^{flox/flox}$	P value
Female BW (g)	21.17 ± 1.55 (n = 7)	20.59 ± 1.24 (n = 5)	0.50
Male BW (g)	26.80 ± 3.32 (n = 6)	27.69 ± 1.84 (n = 8)	0.53

Micro-CT analysis of the femur

	Fgfr1–4$^{flox/flox}$	*Ndrg1-CreERT2;Fgfr1–4*$^{flox/flox}$	P value
BMD (mg/cm^3)	579.6 ± 36.5 (n = 10)	584.9 ± 45.3 (n = 10)	0.78

Pi, inorganic phosphate; Ca, calcium; PTH, parathyroid hormone; 1,25(OH)$_2$D, 1,25-dihydroxyvitamin D; BW, body weight; BMD, bone mineral density; CT, computed tomography.

Supplementary Table S4. Primers used for RT-qPCR. Related to Experimental Procedures, RT-qPCR.

Name	Forward primer	Reverse primer
αKlotho	CCCGATGTATGTGACAGCCAATGG	CTTGGGAGCTGAGCGATCACTAAG
Gapdh	ACCCAGAAGACTGTGGATGG	GGATGCAGGGATGATGTTCT
Cyp27b1	ATGGTGAAGAATGGCAGAGG	TTAGTCGTCGCACAAGGTCA
Cyp24a1	TGGTGCGGATTTCCTTTGT	AGCTGTTTGCGGTCGTCTC
Npt2a	GCTGTCCTCTACCTGCTCGTGTG	GCGTGCCCACTCCGACCATAG
Npt2c	TGTGGGTACTTCGATCACCA	TGTGAGCCAGTTGAAGATGC
Fgfr1	TCACCGCTCTACCTGGAGAT	GGAAGTCGCTCTTCTTGGTG
Fgfr2	GAGTTGCCAGAGGATCCAAA	GACTACTTGCCCGAAGCAAC
Fgfr3	CACCGACAAGGAGCTAGAGG	ACGCAGAGTGATGGGAAAAC
Fgfr4	CGCCAGCCTGTCACTATACAAA	CCAGAGGACCTCGACTCCAA
